# Dynamics of TRF1 organizing a single human telomere

**DOI:** 10.1093/nar/gkaa1222

**Published:** 2020-12-21

**Authors:** Xu Li, Meijie Wang, Wei Zheng, Wei Huang, Zeyu Wang, Kairang Jin, Lin Liu, Zhongbo Yu

**Affiliations:** State Key Laboratory of Medicinal Chemical Biology, College of Pharmacy, Nankai University, 38 Tongyan Road, Tianjin 300350, China; State Key Laboratory of Medicinal Chemical Biology, College of Pharmacy, Nankai University, 38 Tongyan Road, Tianjin 300350, China; State Key Laboratory of Medicinal Chemical Biology, College of Pharmacy, Nankai University, 38 Tongyan Road, Tianjin 300350, China; State Key Laboratory of Medicinal Chemical Biology, College of Pharmacy, Nankai University, 38 Tongyan Road, Tianjin 300350, China; State Key Laboratory of Medicinal Chemical Biology, College of Pharmacy, Nankai University, 38 Tongyan Road, Tianjin 300350, China; State Key Laboratory of Medicinal Chemical Biology, Department of Cell Biology and Genetics, College of Life Sciences, Nankai University, 94 Weijin Road, Tianjin 300071, China; State Key Laboratory of Medicinal Chemical Biology, Department of Cell Biology and Genetics, College of Life Sciences, Nankai University, 94 Weijin Road, Tianjin 300071, China; State Key Laboratory of Medicinal Chemical Biology, College of Pharmacy, Nankai University, 38 Tongyan Road, Tianjin 300350, China

## Abstract

Chromosome stability is primarily determined by telomere length. TRF1 is the core subunit of shelterin that plays a critical role in telomere organization and replication. However, the dynamics of TRF1 in scenarios of telomere-processing activities remain elusive. Using single-molecule magnetic tweezers, we here investigated the dynamics of TRF1 upon organizing a human telomere and the protein-DNA interactions at a moving telomeric fork. We first developed a method to obtain telomeres from human cells for directly measuring the telomere length by single-molecule force spectroscopy. Next, we examined the compaction and decompaction of a telomere by TRF1 dimers. TRF1 dissociates from a compacted telomere with heterogenous loops in ∼20 s. We also found a negative correlation between the number of telomeric loops and loop sizes. We further characterized the dynamics of TRF1 at a telomeric DNA fork. With binding energies of 11 *k*_B_*T*, TRF1 can modulate the forward and backward steps of DNA fork movements by 2–9 s at a critical force of *F*_1/2_, temporarily maintaining the telomeric fork open. Our results shed light on the mechanisms of how TRF1 organizes human telomeres and facilitates the efficient replication of telomeric DNA. Our work will help future research on the chemical biology of telomeres and shelterin-targeted drug discovery.

## Graphical Abstract

**Graphical Abstract:**
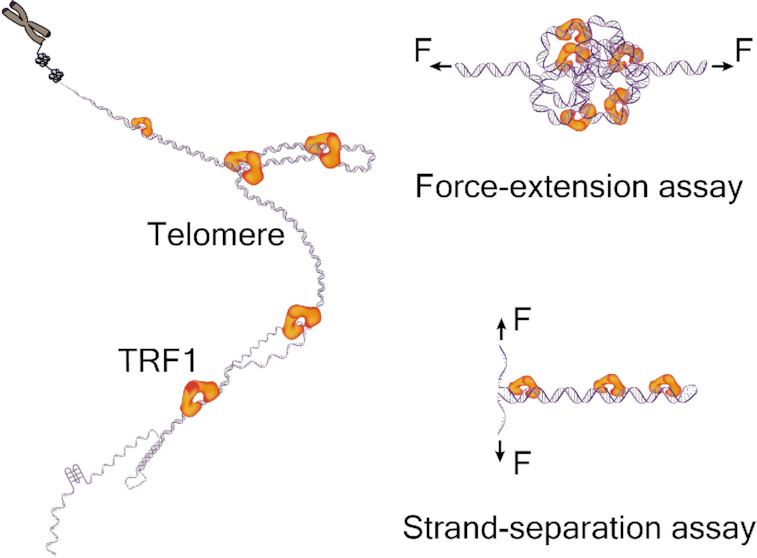
We use single-molecule magnetic tweezers to precisely measure the absolute length of single human telomeres and reveal the dynamics of TRF1 upon organizing a telomere.

## INTRODUCTION

Telomeres protect the termini of human chromosomes (1). Telomere attrition causes maladaptive cellular changes, limiting tissue renewal capacity and leading to aging or human diseases (2). The short telomeres, but not the ones in averaged length, determine cell viability and chromosome stability (3). Telomeres are under tight regulation by telomerase, as well as protected by shelterin proteins (4,5). Shelterin subunits organize telomeres into globular structures through complex interactions with telomeric DNA (6,7). Removal of shelterin triggers chromosome end-protection issues, DNA damages, and fusion of telomeres (8–12).

The core subunits of shelterin have two distinct Myb-related telomeric DNA binding proteins, TRF1 and TRF2 (13,14). Telomeric organization by TRF1 and TRF2 is modulated by nucleosomes (15). The N-terminal domains of TRF1/2 can regulate their capability of condensing telomeric DNA (16). TRF1 bends telomeric DNA as a dimer (17). Self-dimerization of TRF1/2 mediated by a TRFH domain is also essential for compacting telomeres (6,18). Although lacking structural information of full-length protein at atom resolution, evidence from electron microscopy showed that TRF1 adapts a molecular conformation, assisting the DNA binding activity with its TRFH domain accessible to other TRF1 partners (9). More interestingly, TRF1 plays an architectural role at telomeres by recognizing binding sites spaced far apart on DNA independently from orientation or binding sites on two different DNA molecules with extreme spatial flexibility (19,20).

Other than chromosome protection, TRF1 also plays a critical role in sister telomere cohesion (21,22), DNA damage responses (23,24), telomere transcription, and replication (25–29). Replication machinery encounters significant challenges at telomeres due to the TTAGGG repeats, which resemble fragile sites (27). TRF1 is essential for efficient replication by recruiting helicase and shelterin repressor to solve TTAGGG repeat-associated replication issues (28,29). However, TRF1 dynamics in telomere compaction and telomeric fork movement remains elusive.

Super-resolution microscopy has been used in imaging telomere architecture and measuring telomere volume dynamics (6,30–32). The complex scenario in a cell generated controversial observations (6,31,32). Single-molecule assays performed *in vitro* provided well-controlled experimental setups to examine the telomere dynamics upon interacting with shelterin and replication proteins (29,33–35). Single-molecule mechanical tools, e.g. magnetic tweezers, have been used in the research of transcription and replication, providing deep insights into molecular mechanisms of protein-DNA interactions at a fork configuration (36,37).

Using single-molecule mechanical techniques of magnetic tweezers, we here investigated the dynamics of TRF1 organizing a single human telomere and the TRF1–DNA interactions at a telomeric fork upon strand separation. After preparing single human telomeres from K562 cells for single-molecule mechanical assays, we established a precise method for directly measuring the length of a single human telomere. By sampling from a total population of human telomeres, the single-molecule method can reveal the length distribution of telomeres, especially the short ones at defined length ranges. Examining the compaction and decompaction of a single human telomere by TRF1, we found that a TRF1 dimer can compact a single human telomere by interacting with two targeting sites separated far apart. TRF1 dissociates from a compacted telomere with heterogenous loops in ∼20 s. We also found a negative correlation between the number of telomeric loops and loop sizes. We further characterized the dynamics of TRF1 at a telomeric DNA fork using single-molecule strand-separation assays. With binding energies of 11 *k*_B_*T*, TRF1 can modulate the forward and backward steps of DNA fork movements by 2–9 s at a critical force of *F*_1/2_, thereby generating an overall effect of maintaining the telomeric fork at an open state for efficient replication. Our bottom-up methods of single telomere compaction assay and the telomeric fork assay allow us to investigate how shelterin proteins protect chromosomes from a perspective of mechanics and kinetics. Our results shed light on the mechanisms of how TRF1 itself facilitates the efficient replication of a telomere. Our methods and findings will help future research on telomere biology, epidemiology, cancer therapy, as well as shelterin-targeted drug discovery.

## MATERIALS AND METHODS

Other than noted, we have purchased chemicals from Sigma-Aldrich, DNA oligos from Sangon Biotech, and enzymes from New England Biolabs.

### Preparation of single human telomeres for force–extension measurements

We cultured K562 cells (ATCC^®^CCl-243™, ATCC, USA) in RPMI (Roswell Park Memorial Institute) 1640 Medium (Cat#: SH30023.01, Hyclone, USA) supplemented with 10% fetal bovine serum (Cat#: 10099-141, Gibco, USA) at 37°C in a 5% CO_2_ in air atmosphere. At 1 × 10^6^ cells/ml, we collected K562 cells and extracted genome DNA using a commercial kit (DNeasy Blood & Tissue Kit, Cat#: 69506, Qiagen, Germany). Genomic DNA integrity was evaluated by running a 1% agarose gel electrophoresis at 150 V for 30 min with 400 ng of DNA samples, as suggested in the literature (38). DNA samples without a smear in the agarose gel went for restriction enzyme digestion. We first digested 20 μg of K562 DNA with 10 U of CviAII in 50 μl of 1× CutSmart^®^ buffer (NEB, USA) at 25°C for 12 h. We next supplemented the reaction with three more enzymes (BfaI/MseI/NdeI, 10 U of each) and continued the digestion at 37°C for another 12 h. Enzymes were heat-inactivated at 80°C for 20 min. We then filled the 5′ overhangs (TA or AT) of K562 DNA fragments using 5 U of Klenow fragment (3′-5′ exo-) with 1:1 ratio mixture of dATP and digoxigenin-dUTP (Cat#: 11093088910, Roche, Switzerland) at 37°C for 12 h. Klenow reaction thus modified the proximal ends of K562 telomeres with digoxigenin-dUTP for later affinity interactions.

We commercially synthesized biotinylated DNA oligo of biotin-(5′-TAACCC-3′)_3_. The probe can bind telomeres via base-pairing. We then added streptavidin-coated beads (Cat#: 65305, M270, Invitrogen, USA) to capture K562 telomeres via biotin–streptavidin interactions. We isolated the M270 beads with K562 telomeres using magnets and washed other genomic DNA fragments away. The human telomeres immobilized on M270 beads underwent measurements using single-molecule force spectroscopy.

### Dot-blot analysis of biotin-(5′-TAACCC-3′)_3_-enriched telomeric DNA

We checked the biotin-(5′-TAACCC-3′)_3_-enriched telomeric DNA using dot-blot analysis. We mixed the commercially synthesized probe of biotin-(5′-TAACCC-3′)_3_ with the K562 genomic DNA fragments after restriction enzyme digestion. The mixture underwent incubation at 75°C for 3 min, then gradually cooled down to 25°C, facilitating the probe to bind telomeres specifically. We next added streptavidin-coated beads (Cat#: 65305, M270, Invitrogen, USA) with DNA and left at room temperature for 2 h. The beads were then collected using a magnet and washed three times using a buffer of 20 mM HEPES (pH 7.5), 1 mM EDTA, 100 mM NaCl and 0.0063% Tween-20 to remove non-telomeric DNA. We then heated beads at 95°C for 25 min to detach the telomeric DNA from the probe. Using magnets to hold the M270 beads, we recovered the DNA in solution twice. The eluted DNA was further incubated at 95°C for 10 min, and then loaded onto Hybond membranes (Cat#: RPN303B, GE, USA) in 2× SSC buffer containing 0.3 M NaCl and 0.03 M sodium citrate (pH 7.0). We immersed the membranes in a denaturation buffer containing 0.5 M NaOH and 1.5 M NaCl for 10 min. The membranes were then transferred to a neutralization buffer containing 3 M NaCl and 0.5 M Tris–HCl (pH 7.5) for 10 min. DNA was fixed on the membranes by baking at 120°C for 25 min. After submerging the membranes in 2× SSC buffer for 10 min, hybridization was performed either with a digoxigenin-labeled telomeric probe of dig-(5′-CCCTAA-3′)_3_ at 42°C for the detection of telomeric DNA or a digoxigenin-labeled Alu probe of dig-(5′-GTGATCCGCCCGCCTCGGCCTCCCAAAGTG-3′) at 55°C for the detection of non-telomeric sequences. After the washing and blocking steps, probes were detected using an anti–DIG-AP antibody (Cat#: 11093274910, Roche, Switzerland) on a chemiluminescent imaging system (Tanon-5200, Tanon Science & Technology Co., Ltd., Shanghai, China). Image analysis was done in ImageJ (NIH Image, USA).

### Preparation of telomeric hairpin constructs for strand-separation assays

Our hairpin constructs consist of general parts for mechanical manipulation and unique telomeric sequences. The stem contains either 23 (long) or 13 (short) repeats of TTAGGG. The general parts are two handles, a junction, and a loop. To prepare handles for mechanical manipulation, we ran PCR to prepare DNA fragments of ∼676 bp using a template of pBluescript II SK(+) (Cat#: 212205, Agilent, USA) and a dNTP mixture supplemented with biotin-16-dUTP or digoxigenin-11-dUTP (Cat#: 11093070910 or 11093088910, Roche, Switzerland) (Forward and reverse primers for handles in Supplementary Table S1). We mixed dTTP with either biotin or digoxigenin modified dUTP at a molar ratio of 10:1. We used restriction sites of BbvCI or PpuMI for ligation between two handles and a hairpin junction. The assembly of the hairpin junction used four DNA oligos (Junction oligos in Supplementary Table S1), which were annealed at equimolar ratios in 10 mM Tris–HCl (pH 8.0) with a temperature program of heating to 95°C for 3 min, then reducing 0.1°C per step (700 steps in total) and finally cooling down to the room temperature of 25°C. We ligated the handles and the hairpin junction with a molar ratio of 5:1:5 (biotin:junction:digoxigenin) at 16°C for 12 h.

To prepare the long hairpin stem with 23 telomeric repeats, we first annealed ssDNA oligos (Stem oligos in Supplementary Table S1) using the temperature program the same as above. We also prepared the hairpin loop (Loop 1 oligo in Supplementary Table S1) using the same method. With cohesive end termini, we ligated the telomeric stem and the loop with a molar ratio of 1:5:10 (Stem 1/1c: stem 2/2c: loop 1) at 16°C for 12 h. Finally, the intermediate products were ligated to obtain the hairpin construct with 23 telomeric repeats at 16°C for 10 h, followed by 25°C for 2 h.

To prepare the short hairpin construct with 13 telomeric repeats, we performed one-pot ligation using the biotin handle, the digoxigenin handle, the hairpin junction, the telomeric stem 1/1c, and the hairpin loop 2 at a molar ratio of 5:5:1:10:10 (Supplementary Table S1).

Final products were purified using 1% agarose and stored at −20°C.

### Expression and purification of TRF1

We molecularly cloned human TRF1. We first obtained TRF1 coding sequences from a plasmid (Cat#: 53209, Addgene, USA) by PCR (see primers in Supplementary Table S1). With restriction enzymes of BamHI and XhoI, we prepared TRF1(3–439) DNA (see protein sequences in Supplementary Table S2), which were purified using 1% agarose gel and a gel extraction kit (Cat#: D2500-02, Omega Bio-tek, Inc., USA). We next cloned the TRF1 sequence into a vector of modified pET32a.

We expressed TRF1 in *Escherichia coli* BL21(DE3). The LB medium contains 100 μg/ml of Ampicillin sodium for culturing. After induction for 18 hours with 0.5 mM of isopropyl-β-d-1-thiogalactopyranoside (IPTG) at 25°C, we collected cell pellets by centrifugation which was followed by resuspension in a lysis buffer (50 mM Tris–HCl pH 8.0, 400 mM NaCl, 5% glycerol) supplemented with 20 U RQ1 RNase-Free DNase (Cat#: M6101, Omega Bio-tek, Inc., USA) and 5 μg/ml RNase A (Cat#: R4875, Sigma-Aldrich, USA). Cell lysis was facilitated by sonication with a 50-min program of repeating 1-s-on and 3-s-off using ultrasonic cell crusher (Scientz-IID, SCIENTZ, China). We then collected supernatant by ultracentrifugation at 12 000 rpm. We continued to incubate the supernatant with Ni-NTA agarose beads (Cat#: 17526801, GE Healthcare, USA) for 1 h at 4°C. The following washing step used the lysis buffer supplemented with 20 mM imidazole. To remove the trx tag in TRF1, we used 3C protease (Cat#: 88946, ThermoFisher, USA).

We used ion-exchange chromatography and size exclusion chromatography to improve the purity of proteins further. Protein solution first went through HiTrap Heparin HP (Cat#: 17–0407-01, GE, USA). Next, we performed the gel filtration chromatography on Superdex 200 Increase 10/300 GL (Cat#: 28-9909-44, GE, USA), which was equilibrated with 20 mM Tris–HCl (pH 8.0) and 200 mM NaCl. The purified proteins were concentrated to 2 mg/ml and stored at −80°C.

### Electrophoretic mobility shift assay

We prepared a telomeric dsDNA for Electrophoretic mobility shift assay (EMSA) from two oligodeoxynucleotides, GTTAGGGTTAGGGTTAG and FAM-CTAACCCTAACCCTAAC, which contain two TTAGGG sites. Two oligodeoxynucleotides were mixed at an equal molar ratio, heated to 95°C for 30 seconds, and slowly cooled down to 25°C in 2 h. In a buffer containing 20 mM HEPES (pH 7.5), 100 mM NaCl, 1 mM EDTA, and 0.0063% Tween-20, we titrated 10 nM of the telomeric dsDNA with TRF1 from 0 to 200 nM (Dimer concentration). The reaction underwent 30 min on ice. We then loaded 20 μl of the reaction mixture to 5% PAGE gel, which ran at 4°C and 50 V for 100 min in 1× TBE (Tris–borate–EDTA) buffer. We took the gel images at 495 nm illumination in an imaging system (PXi9, Syngene, UK). Image analysis was done in ImageJ (NIH Image, US).

### Terminal restriction fragment analysis of telomere length

We performed terminal restriction fragment (TRF) analysis using components from the kit of *T*elo*TAGGG*™ Telomere Length Assay (Cat#: 12209136001, Roche, Switzerland). The combination of restriction enzymes used here is CviAII/NdeI/MseI/BfaI instead of HinfI/RsaI in the kit. Telomere length (TL) assay has been done at room temperature other than specifically noted. The genomic DNA of K562 cells digested by CviAII/NdeI/MseI/BfaI enzymes underwent electrophoresis in 0.8% agarose gel at 5 V/cm for 4 h. Next, DNA was transferred from the gel to a positive-charged nylon membrane by Southern blotting. The transferred DNA was then fixed by baking the membrane at 120°C for 25 min. We hybridized the membrane with a digoxigenin-labeled telomere probe at 42°C for 10 h. After hybridization, the membrane underwent washing twice with buffer I (2× SSC, 0.1% SDS) for 5 min and twice with prewarmed buffer II (0.2× SSC, 0.1% SDS) at 50°C for 30 min. With one more washing step in 1× washing buffer for 5 min, we put the membrane in 1× blocking solution for 1 h. After an incubation step with an anti-digoxigenin antibody in 1× blocking solution for 4 h, we washed the membrane twice with 1× washing buffer for 20 min. We then incubated the membrane in a 1× detection buffer for 5 min. After discarding the detection buffer, the membrane underwent incubation with CDP-star for 10 min. We finally visualized the telomere probe using Tanon-5200 Chemiluminescent Imaging System (Tanon Science & Technology Co., Ltd., Shanghai, China). TL quantification was done using the software of TeloTool (39).

### Single-molecule assays using smMT

We used homemade single-molecule magnetic tweezers (smMT), like previously described devices (40–42). Taking advantage of a setup of microsphere-DNA-coverslip, we ran assays on the smMT in a reaction chamber. We routinely mixed 20 μl of streptavidin-coated beads (Cat#: 65305, M270, Invitrogen, USA) with constructs of DNA hairpins or human telomeres in a volume of 40 μl containing 20 mM of HEPES (pH 7.5), 1 mM of EDTA, 100 mM of NaCl and 0.0063% Tween-20. The coverslip surface to support single-molecule assays has a matrix of nitrocellulose (0.1%, m/v). On the nitrocellulose matrix we next incubated anti-digoxigenin antibody (0.1 mg/ml, Cat#: 11093274910, Roche) for 0.5–2 h and passivated with BSA (5 mg/ml) overnight. The surface with antibodies can immobilize DNA, which was already bound to beads. At a sampling rate of >100 Hz, we ran constant-force, force–ramp and force–jump assays in a buffer containing 20 mM of HEPES (pH 7.5), 1 mM of EDTA, 100 mM of NaCl and 0.0063% Tween-20.

In force–jump assays, we developed a force–manipulating protocol to examine how TRF1 compacted a single human telomere of dsDNA. We first allowed interactions between TRF1 and a single human telomere at a resting force (*F*_rest_). We next increased forces from *F*_rest_ to a testing force (*F*_test_), which should be strong enough to break the interactions between proteins and DNA and generate a series of distinct steps. To assure the complete rupture of the protein–DNA complex, we then strengthened *F*_test_ to strong forces (*F*_high_ and *F*_max_). We finally reversed the mechanical manipulations by sequentially decreasing the forces from *F*_max_ to *F*_high_, *F*_test_ and *F*_rest_. By adjusting the durations of forces, we can investigate the kinetics of molecular interactions.

We used a similar protocol of force jumps as above to manipulate constructs of hairpin DNA. DNA extensions are short at a low force (*F*_low_), which is less than the critical forces of unfolding a hairpin. In 390 ± 30 ms (mean ± SD, *n* = 10), we increased the force from *F*_low_ to a testing force (*F*_test_) where DNA extensions become long, i.e. the hairpin is fully open. TRF1-binding events interrupt the unfolding of a hairpin, resulting in pauses at intermediate extensions. We next increased *F*_test_ to a higher force (*F*_high_) where the DNA further extends. We then decreased the force from *F*_high_ to *F*_test_, which serves for reference purposes. We finally lowered down the force to *F*_low_, completing a circle of our force protocol. By optimizing the *F*_low_, *F*_test_ and *F*_high_, as well as their durations, we can tune a force protocol according to the tested proteins and hairpin constructs (37,43–46).

The probabilities of observing binding signals on the tested DNA molecules are 38%, 50%, 76% and 60% for [TRF1] = 10, 20, 25 and 40 nM, respectively.

### Analysis of single-molecule data

We analyzed all the data from smMT in MatLab (R2017a, Mathworks, US).

For force–extension (*F*–*x*) measurements of single human telomeres, we convert the extension at 17 pN from nanometers to base pairs (bp) using a factor of 0.33 nm/bp, which is from an extensible Worm-Like-Chain (WLC) at the ionic strength close to our conditions (100 mM NaCl) (47),

(1) $$\begin{equation*}\frac{x}{{L_c^{ds}}} = 1 - \frac{1}{2}{\left( {\frac{{{k_B}T}}{{FL_p^{ds}}}} \right)^{\frac{1}{2}}} + \frac{F}{S}\end{equation*}$$

where $L_p^{ds}$ of 51.1 nm is the dsDNA persistence length, $L_c^{ds}$ the dsDNA contour length, *S* of 1006 pN the stretch modulus and *k*_B_*T* for that Boltzmann's constant times temperature.

Using a step-fitting algorithm (48), we measured the pausing time at a specific position in traces of strand-separation assays. For hopping traces from constant-force assays, we used an algorithm of hidden Markov modeling (HMM) to obtain states of positions and corresponding time durations (49,50). Because data were collected at a minimum of 100 Hz, we only took the pausing time >20 ms considering the Nyquist frequency.

To measure the pausing positions when unfolding a hairpin construct in strand-separation assays, we performed zero-correction for each trace by subtracting the bead height where the hairpin fully opened at the force of *F*_test_. After zero-correction at *F*_test_, the resulting change in extensions represents the length of ssDNA released while unfolding a hairpin. In a histogram for the extension of each trace, peaks at specific extension indicate the blockage of hairpin opening due to TRF1 binding.

To theoretically calculate the conversion factor from nanometer to nucleotide (nt) regarding forces as a function of extensions, we employ the Marko-Sigga equation of a WLC model (51),

(2) $$\begin{equation*}F = \frac{{{k_B}T}}{{L_p^{ss}}}\left( {\frac{1}{4}{{\left( {1 - \frac{x}{{L_c^{ss}}}} \right)}^{ - 2}} + \frac{x}{{L_c^{ss}}} - \frac{1}{4}} \right)\end{equation*}$$

where $L_p^{ss}$ is the ssDNA persistence length, $L_c^{ss}$ the ssDNA contour length, *k*_B_ the Boltzmann constant and *T* the temperature. For ssDNA at the salt conditions of 100 mM NaCl, we take $L_p^{ss}$ = 0.87 nm and $L_c^{ss}$ = 0.69 nm/nt, as suggested by Bosco *et al.* (52).

## RESULTS

### Length measurements of single human telomeres using magnetic tweezers

**Figure 1.**
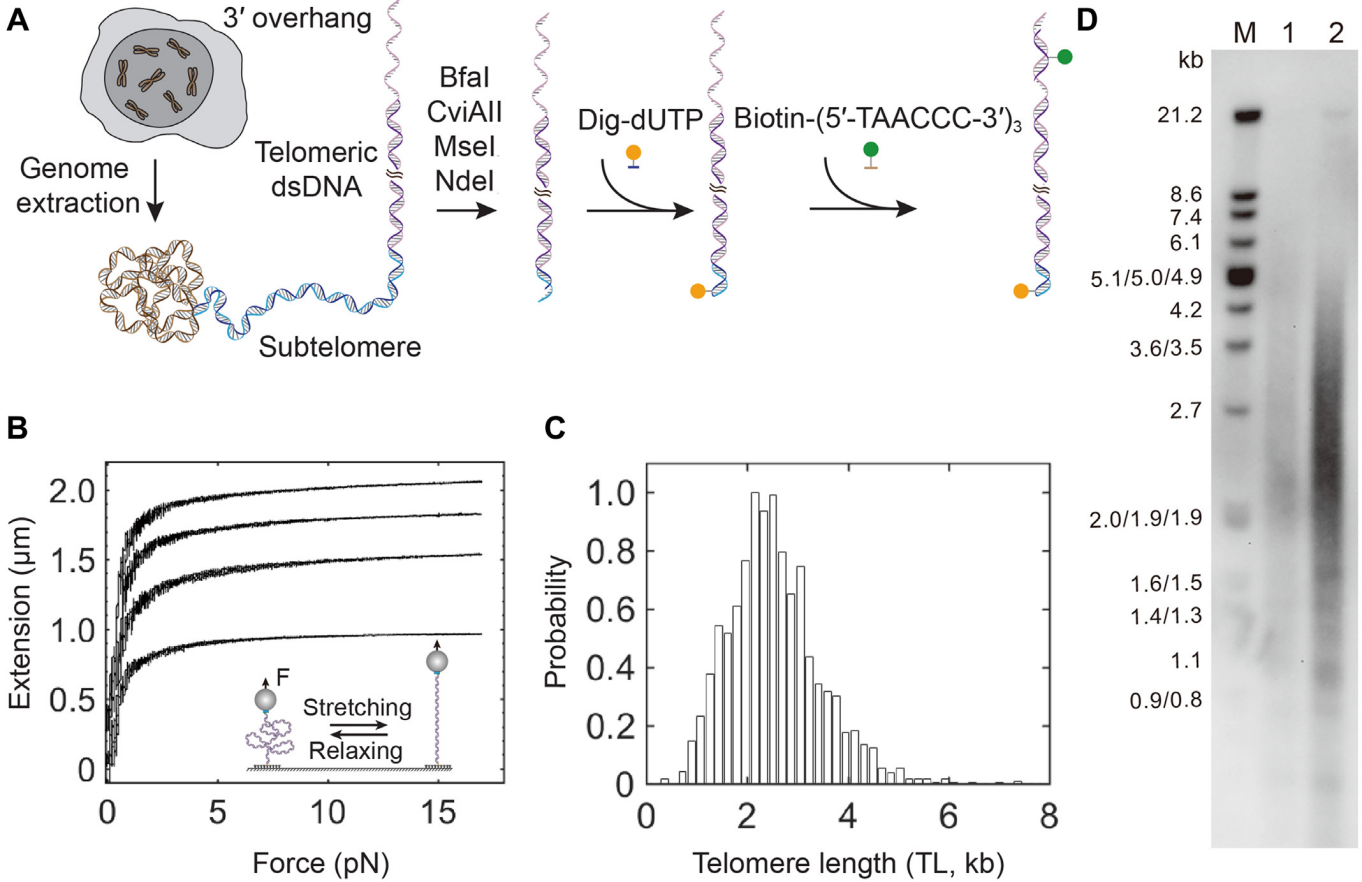
Preparation of single human telomeres and length measurements using force–extension assays. (**A**) Scheme of single telomere preparation. The genome of K562 cells underwent digestion by four restriction enzymes, BfaI, CviAII, MseI, and NdeI. Genome digestion generates AT or TA overhangs in the residual subtelomere. Digoxigenin-modified dUTP (Orange sphere) fills in the AT or TA overhangs via Klenow reaction. Biotin-modified (5′-TAACCC-3′)_3_ oligos (Green sphere) recognize the telomeric ssDNA overhang by base pairing. Digoxigenin and biotin enable a single human telomere to interact with glass slide treated by anti-digoxigenin antibody and streptavidin-coated beads, respectively. (**B**) Telomere length measurements by force–extension assays. In a reaction chamber of single-molecule magnetic tweezers containing a buffer of 20 mM of HEPES (pH 7.5), 1 mM of EDTA, 100 mM of NaCl and 0.0063% Tween-20 at 23°C, two surfaces of a glass slide and a microsphere immobilized a single human telomere via digoxigenin–antibody and biotin–streptavidin affinity interactions (cartoon). Using single-molecule force spectroscopy, four typical force–extension curves of single human telomeres show distinct length responses to forces ranged between 0 and 17 pN with a force loading rate of ±4 pN/s. (**C**) Length distribution of single human telomeres from K562 leukemia cells measured by single-molecule magnetic tweezers. The distribution shows a length with 2.5 ± 0.9 kb (mean ± SD, *n* = 1975). The minimum and maximum lengths are 0.3 and 7.4 kb, respectively. (**D**) Terminal restriction fragment (TRF) analysis using CviAII/NdeI/MseI/BfaI enzymes. Marker sizes are noted in the M lane. Lane 1 and lane 2 contain 2 μg and 4 μg DNA, respectively. The estimated TL is 2.7 ± 2.3 kb based on lane 2 using the software of TeloTool (39).

To investigate the single-molecule behavior of TRF1 organizing human telomeres, we first prepared single human telomeres for mechanical manipulations. We extracted genomic DNA from K562 leukemia cells (Figure 1A), a standard cell line for telomere length (TL) assessment (53). We assessed the integrity of DNA using agarose gel electrophoresis, which revealed intact genomes without degradation (Supplementary Figure S1). The K562 genomic DNA then underwent digestion by four restriction enzymes (BfaI/CviAII/MseI/NdeI), which have been used in terminal restriction fragment (TRF) analysis to measure TL (54). The resulting terminal restriction fragments contain full telomeres from all chromosomes with minimized subtelomeric regions. The four restriction enzymes generate either ‘TA’ or ‘AT’ 5′ overhangs at the proximal end of telomeres. To achieve affinity interactions in single-molecule mechanical manipulations, we used the Klenow fragment of DNA polymerase I to incorporate digoxigenin-dUTP in the 5′ overhangs of ‘TA’ or ‘AT’ to form blunt ends. Because the Klenow fragment had neither 5′ to 3′ nor 3′ to 5′ exonuclease activity, the 3′ overhangs of telomeres with TTAGGG repeats remained intact. Therefore, we designed the biotinylated DNA oligos of Biotin- (5′-TAACCC-3′)_3_ to capture the 3′ overhangs of telomeres. With biotin and digoxigenin modifications on K562 telomeres, we were able to manipulate single human telomeres using magnetic tweezers mechanically.

We also performed a dot-blot analysis to check the background of non-telomeric DNA after enrichment using the telomere-specific probe of biotin-(5′-TAACCC-3′)_3_ (Supplementary Figure S2, Materials and methods). The recovery of telomeric DNA is 96% ± 5% (mean ± SD, *n* = 3). In addition, the telomeric DNA is 3298 ± 2269 times more than the non-telomeric Alu-repeat DNA after enrichment (mean ± SD, *n* = 3). Thus, the background of non-telomeric DNA in our TL measurements is negligible.

We next performed force–extension measurements of TL using single-molecule magnetic tweezers. Biotin and digoxigenin modified telomeres form affinity interactions with streptavidin-coated superparamagnetic beads and anti-digoxigenin antibody covered glass, respectively (Figure 1B, cartoon). By modulating the strength of a magnetic field, magnetic tweezers can exert forces on the beads, hence the single human telomeres. In a force–ramp mode, we stretched and relaxed the single telomeres with a loading rate of ±4 pN/s. TL responds to forces as a function of the WLC (51,55). We can thus employ the WLC model-assisted single-molecule ruler to precisely measure the length of individual telomeres (47,55–59), which is in contrast to the rough estimation used by conventional methods of gel electrophoresis or fluorescence analysis (38,60–62). Force–extension traces of single telomeres are distinct in length, quantitatively revealing how TL varies among chromosomes. The TL distribution measured by force–extension assays centers at 2.5 ± 0.9 kb (mean ± SD, *n* = 1975) (Equation 1 and Figure 1C). We performed terminal restriction fragment (TRF) analysis, a golden standard of TL measurements, as a control for our single-molecule method (Materials and methods). We prepared the terminal restriction fragments using the same set of enzymes as above. The TRF analysis revealed the TL of K562 cells to be 2.7 ± 2.3 kb (Figure 1D). Our single-molecule TL measurements are consistent with the results by TRF analysis and that in the literature (54). We thus established a method to manipulate single human telomeres mechanically.

### TRF1 dissociated from a compacted telomere with heterogenous loops in 23 s

**Figure 2.**
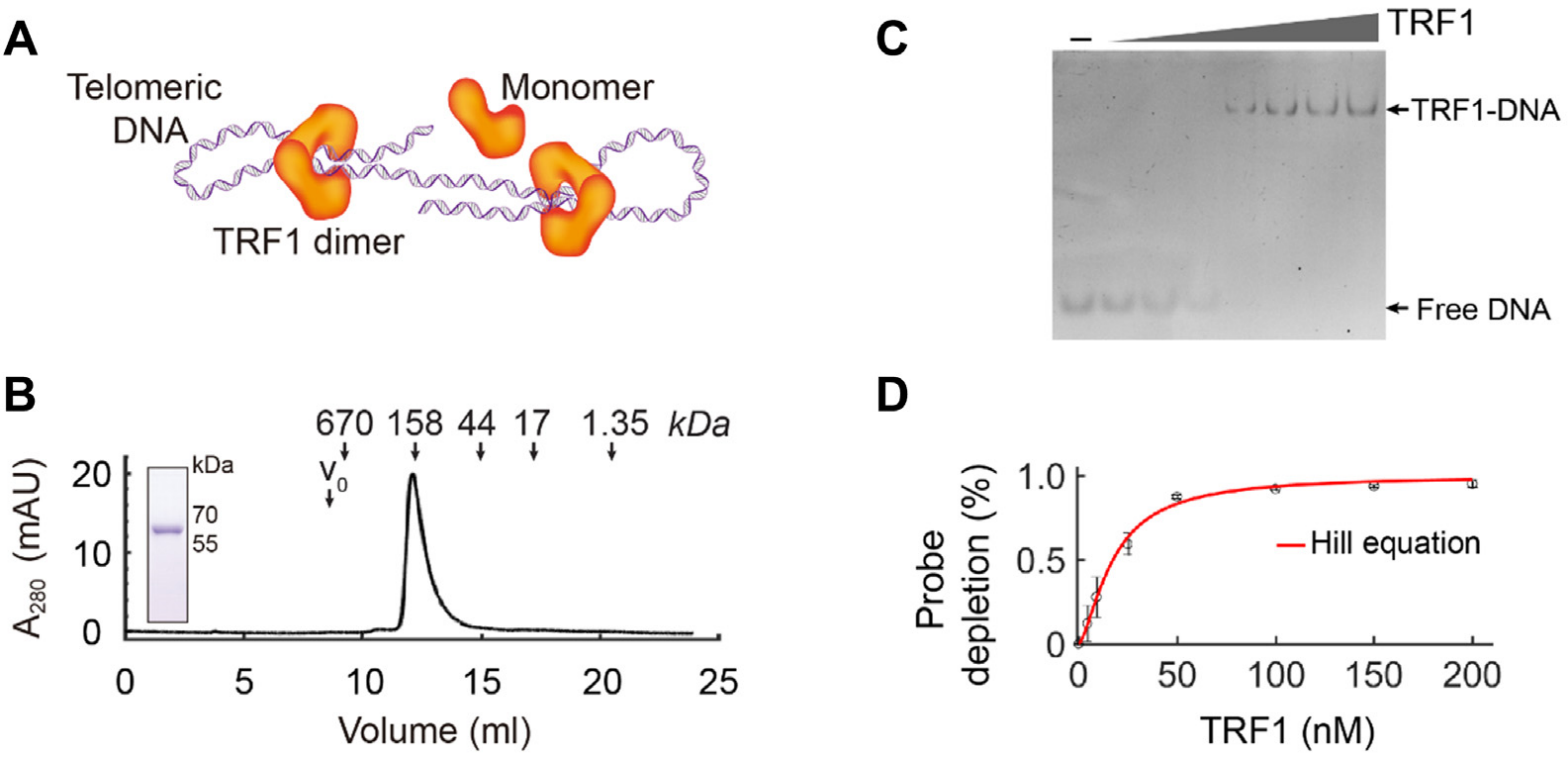
Preparation of TRF1 and the activity measurement using EMSA. (**A**) The scheme shows that TRF1 dimer can compact a telomeric dsDNA. (**B**) Human TRF1 protein after purification is in a high homogeneity, as shown by both size exclusion chromatography and SDS-PAGE (12%, stained in Coomassie Blue). The size exclusion chromatography analysis reveals that TRF1 exists as a dimer configuration. Downward arrows indicate the elution volumes of standard molecular markers. (**C**) Electrophoretic mobility shift assay (EMSA). Purified TRF1 (0–200 nM) was incubated with 10 nM of FAM-labeled telomeric dsDNA, CTAACCCTAACCCTAAC and electrophoresed in 5% PAGE gel as shown. Free DNA and TRF1–DNA bands are indicated. (**D**) The telomeric dsDNA binding affinity of the purified TRF1 shows a *K*_d_ of 17.5 ± 0.7 nM by fitting the Hill equation (red curve) to the EMSA data (mean ± SD, *N* = 3).

To examine the dynamics of TRF1 compacting single human telomeres, we molecularly cloned and purified human TRF1. TRF1, directly interacting with telomeric DNA (13), contains a C-terminal domain (Myb) for binding dsDNA (Double-stranded DNA) and an N-terminal domain (TRFH) for self-dimerization (18,63). The dimerization of TRF1 plays an essential role in shelterin compacting telomeric chromatin (6,9) (Figure 2A). We expressed the recombinant protein of TRF1 in *E. coli* cells (Materials and methods). After removing the affinity tag, the purified protein shows homogeneity with >95% purity by SDS-PAGE (Figure 2B, inset). Using size exclusion chromatography, we assessed the molecular weight of the purified TRF1, indicating that the protein forms a dimer (Figure 2B). To measure the binding affinity (*K*_d_), we performed the electrophoretic mobility shift assay (EMSA) (Materials and methods, Figure 2C). Estimation using the Hill equation (44) gives a *K*_d_ of 17.5 ± 0.7 nM (mean ± SE, *n* = 3, Figure 2D). We use dimer concentrations for TRF1 throughout this work. The *K*_d_ is lower than the physiological concentration of TRF1, which is estimated to be 21.6 nM (12.2–43.7 nM) using the number of TRF1 in a Hela cell (40 000 molecules/HeLa cell) (64) and the Hela cell volume of 1540 μm^3^ (760–2730 μm^3^) (65).

**Figure 3.**
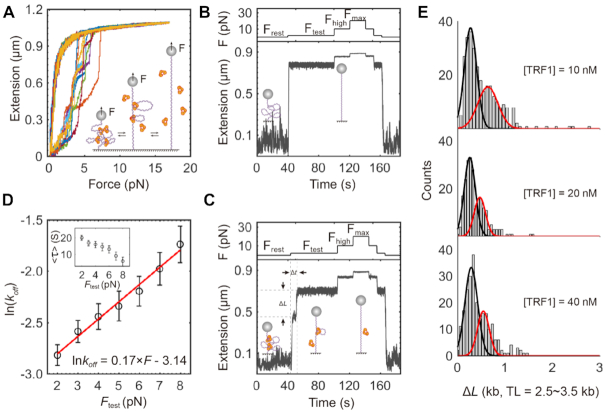
Rupture of protein-dsDNA complexes between TRF1 and a single human telomere upon forces. (**A**) TRF1-telomere complexes rupture in force–ramp assays. At the protein concentration of [TRF1] = 10 nM, protein–dsDNA complexes repetitively rupture and form when forces oscillate between 0 and 17 pN at a loading rate of ±4 pN/s (cartoon). Force–extension traces (*N* = 10) show that rupture events occur as multiple leaps in extension and finish at a stretching force <8 pN in a buffer of 20 mM of HEPES (pH 7.5), 1 mM of EDTA, 100 mM of NaCl and 0.0063% Tween-20 at 23°C. Upon relaxing, the extension of a single telomere smoothly responds to forces without distinguishable refolding steps. (**B**) Force–jump assays show the responses of a single human telomere extension to forces in real-time. The applied protocol of forces is *F*_rest_ = 0 pN, *F*_test_ = 2–8 pN, *F*_high_ = 10 pN and *F*_max_ = 20 pN. Buffer and temperature conditions are the same as those in (A). Sampling rate = 200 Hz. (**C**) Measurements of dissociation time and change in extension upon a rupture event of TRF1–telomere complexes. At the concentration of [TRF1] = 10 nM, force–jump assays reveal rupture events of TRF1-telomere complexes at *F*_test_ after an incubation time of 120 s at *F*_rest_ = 0 pN (cartoon). The duration between two subsequent changes in extension at *F*_test_ indicates the dissociation time of a TRF1 from a single human telomere. *F*_high_ and *F*_max_ assure the complete extension of a single telomere. Buffer and temperature conditions are the same as those in (A). Sampling rate = 200 Hz. (**D**) Dissociation rates of TRF1 from a single telomere as a function of tested forces. The logarithm of the dissociation rate at tested forces (mean ± SD, *n* = 206 from 24 molecules) follows the model of Kramer Bell-Evans (red curve and legend equation). Inset shows the averaged dissociation time $( {\langle \tau \rangle } )$ at *F*_test_. (**E**) Distribution of change in extension upon rupture events of TRF1–telomere complexes. Changes in extension (Δ*L*) were measured using telomeres with TL = 2.5–3.5 kb at [TRF1] = 10, 20 and 40 nM. Black and red curves represent Gaussian fittings.

Force–extension measurements of single human telomeres at the presence of TRF1 proteins reveal DNA compaction events. With TRF1 of 10 nM in a buffer containing 20 mM of HEPES (pH 7.5), 1 mM of EDTA, 100 mM of NaCl and 0.0063% Tween-20, we set out to run force–ramp assays at a force span of 0–17 pN using single-molecule magnetic tweezers (Figure 3A, inset). Extension of a single telomere shows multiple leaps upon stretching (Figure 3A). Comparing to the smooth response of telomere extension to forces at the absence of TRF1 (Supplementary Figure S3), multiple leaps of DNA extension in force–ramp assays provide direct evidence of telomere compaction by TRF1. The rupture of a compacted telomere by 10 nM of TRF1 usually completes at a stretching force <8 pN.

To quantitatively examine how TRF1 compacts a single human telomere, we performed force–jump assays using magnetic tweezers. We modulated forces at four levels, a resting force (*F*_rest_), a testing force (*F*_test_), a high force (*F*_high_) and a maximum force (*F*_max_) (Materials and methods, Figure 3B). At *F*_rest_ = 0 pN, double-stranded telomere DNA can randomly coil in the buffer, allowing TRF1 protein dimers to bind for compaction. At *F*_test_ = 2–8 pN, the telomeric complex of protein–dsDNA ruptures, releasing the compacted dsDNA. Strong forces of *F*_high_ = 10 pN and *F*_max_ = 20 pN further extend the dsDNA to assure the full extension of a single telomere. We repetitively run the protocol to probe the telomere compaction events at [TRF1] = 10 nM. Because we prepared single telomeres from K562 cells, extensions at a specific force are different among single telomeres, indicating the varied lengths of telomeres from independent chromosomes. By comparing telomeric extensions with or without TRF1, we directly observed rupture events of TRF1–telomere complexes in force–jump assays (Figure 3B versus C).

We can also directly measure the dissociation time upon a rupture event at *F*_test_. The dissociation time before each rupture event, $\Delta t$, indicated the kinetic barrier and local stability for TRF1 unbinding. Kramer Bell-Evans model says that the kinetic rate $k$ for dissociation is a function of force *F*, i.e., $k = \frac{1}{{\langle \tau \rangle }} = {k_0}{\rm{exp}}( {\frac{{F{x^\dagger }}}{{{k_B}T}}} )$, where ${x^\dagger }$ is the activation distance from the TRF1 bound state to the transition state, and ${k_0}$ is the extrapolated TRF1 dissociating rate at *F* = 0 pN. At [TRF1] = 10 nM, we measured the dependence of the averaged dissociation time, $\langle \tau \rangle$, at various *F*_test_ (Supplementary Figure S4 and Figure 3D, inset). Evaluated by Kramer's model above, we found that the logarithm of $k$ linearly responds to the testing forces of 2–8 pN (Figure 3D, red). The estimated ${x^\dagger }$ is 0.7 nm, ∼ 2 bp between the TRF1 bound telomeric complex and the activated state for a rupture event. The dissociation rate $k$ at zero force is 0.04 s^−1^, corresponding to 23 s for the dissociation time upon a rupture event at *F* = 0 pN.

Distribution of changes in extension discloses that TRF1 condenses a single telomere with heterogenous loops. We measured the changes in extension (Δ*L*) at *F*_test_ = 2–20 pN and TL = 2.5–3.5 kb (Equation 1 and Figure 3E). The Δ*L* distributions reveal two major components upon the disruption of TRF1–TRF1 interactions or TRF1-DNA interactions at [TRF1] = 10, 20 and 40 nM. The short Δ*L* of ∼250 bp is independent of the TRF1 concentrations. On the other side, the size of long Δ*L* decreases from 640 ± 212 bp at [TRF1] = 10 nM to 470 ± 120 bp at [TRF1] = 20 nM or 545 ± 124 bp at [TRF1] = 40 nM (Gaussian center ± sigma). Furthermore, Δ*L* of >2000 bp occurs with low frequencies at [TRF1] = 10 nM but is not detectable at [TRF1] = 20 and 40 nM. Together, these data suggest that TRF1–telomere complexes form heterogenous loops, in which the long loop sizes respond to changes of TRF1 concentrations.

### The number of telomeric loops and the loop sizes are negatively correlated

**Figure 4.**
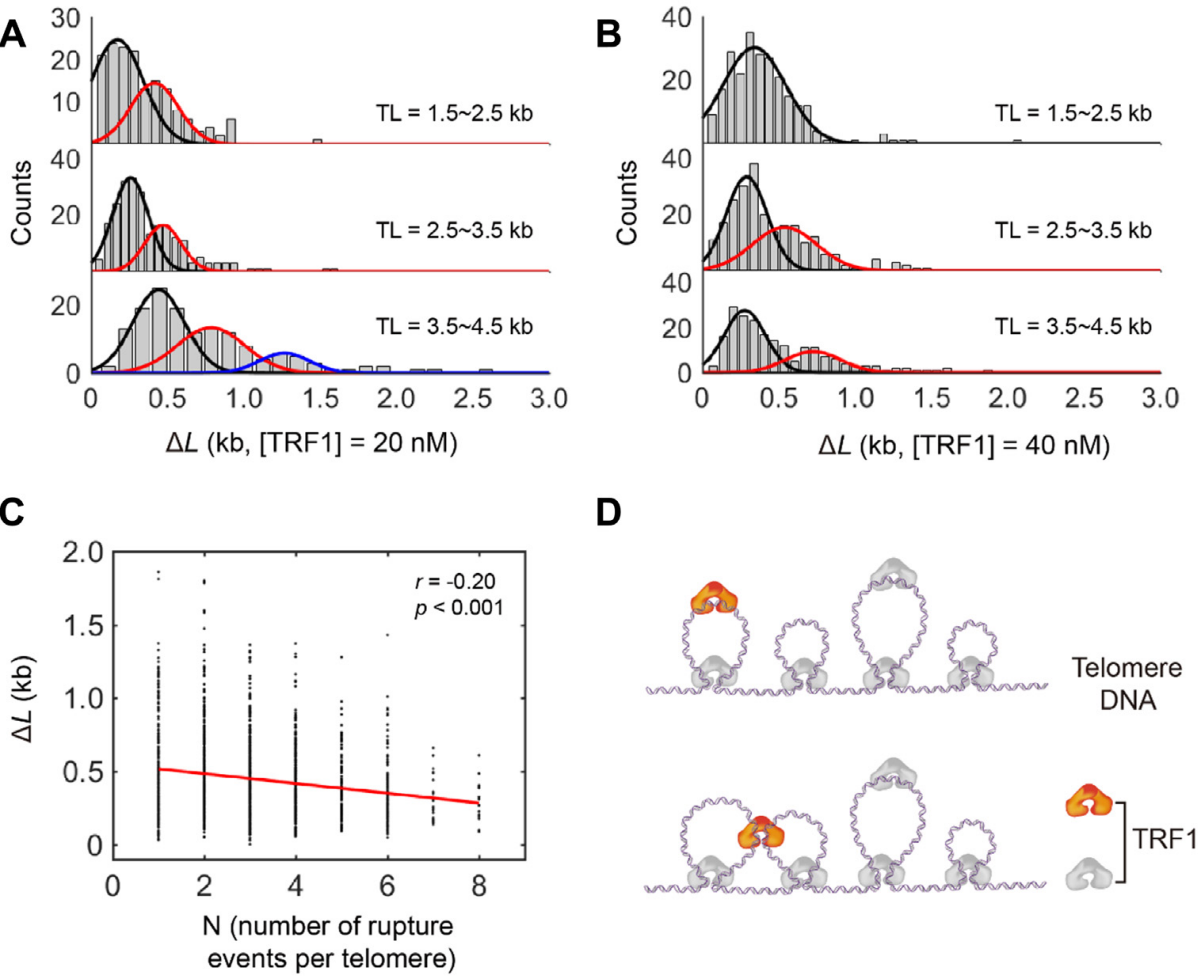
Correlation between the number of telomeric loops and loop sizes in a TRF1-compacted telomere. (**A**) The TL-dependency of telomeric loop sizes (Δ*L*) at [TRF1] = 20 nM. At TL of 1.5–2.5 kb (Top), the Gaussian distributions of Δ*L* are centered at 172 ± 170 and 415 ± 157 bp (*N* = 161 from 29 molecules). At TL of 2.5–3.5 kb (middle), the Gaussian centers shift to 258 ± 117 and 470 ± 120 bp (*N* = 232 from 10 molecules). At TL of 3.5–4.5 kb (bottom), the Gaussian centers are at 442 ± 171, 785 ± 223 and 1266 ± 164 bp (N = 151 from 14 molecules). Curves represent Gaussian fittings. Errors are standard deviations estimated from Gaussian fittings. (**B**) The TL-dependency of Δ*L* at [TRF1] = 40 nM. The Gaussian distributions of Δ*L* are centered at 340 ± 207 bp (top, *N* = 243 from 27 molecules); 290 ± 134 and 538 ± 212 bp (middle, *N* = 238 from 23 molecules); 277 ± 135 and 725 ± 181 bp (bottom, *N* = 203 from four molecules). TL is noted at each panel. Curves represent Gaussian fittings. Errors are standard deviations estimated from Gaussian fittings. (**C**) Correlation between Δ*L* and the number of rupture events per telomere, *N*. The zero-order correlation *r* = –0.20 with *P* < 0.001 (Sample size = 1496 from 131 molecules). (**D**) The cartoon illustrates a possible mechanism that explains the negative correlation between Δ*L* and *N*, i.e. TRF1 can compact a single telomere with primary loop domains to a high-order topology.

Because TL varies from one molecule to another in our experiments, we next checked whether Δ*L* distributions depend on the length of telomeres. We examined the Δ*L* distributions as a function of TL at [TRF1] = 20 and 40 nM (Figure 4A and B). We grouped the telomeres into three categories of TL, i.e. TL = 1.5–2.5 kb, TL = 2.5–3.5 kb, and TL = 3.5–4.5 kb. Δ*L* distributions generally show two or three major peaks except that for TL = 1.5–2.5 kb at [TRF1] = 40 nM, suggesting that heterogenous loops in a telomere are a common feature as that for TL = 2.5–3.5 kb at [TRF1] = 10 nM (Figure 3E versus Figure 4A and B). At [TRF1] = 20 nM, Δ*L* distributions show that the peak centers shift to the longer range with the increment of TL (Figure 4A). Moreover, the Δ*L* distribution shows one more peak for TL = 3.5–4.5 kb than that for shorter TLs, indicating that TL plays a role in the heterogeneity of the TRF1-compacted telomeric loops. Such phenomena were also observed at [TRF1] = 40 nM (Figure 4B). TRF1 at a high concentration of 40 nM tightly compacts a short telomere with a single peak of Δ*L* distribution for TL = 1.5–2.5 kb. When TL becomes longer, Δ*L* distributions show one more peak of longer extensions at [TRF1] = 40 nM. These data suggest that telomeric loop sizes depend on both TRF1 concentration and TL.

Furthermore, we investigated correlations among pairs of TRF1 concentration, TL, Δ*L* and the number of rupture events. We first quantified the number of rupture events per telomere, *N*, which reflects the number of loops tied by multiple TRF1 proteins on a single telomere. We next examined pairwise Pearson's correlations (Supplementary Figure S5). *N* is negatively correlated to Δ*L* (*R* = –0.20, *P* < 0.001, Figure 4C) but positively correlated to TL (*R* = 0.12, *P* < 0.001) and TRF1 concentration (*R* = 0.12, *P* < 0.001). Δ*L* is positively correlated to TL (*R* = 0.27, *P* < 0.001) but shows negative weak correlation with TRF1 concentration (*R* = –0.08, *P* < 0.01). When controlling either TRF1 concentration or TL, the first-order partial correlation between *N* and Δ*L* reveals either a similar relationship (*R* = –0.19, *P* < 0.001) or a stronger one (*R* = –0.24, *P* < 0.001) than that of zero-order, respectively (Supplementary Table S3). Such comparison indicates that tested protein concentrations contribute less than TL to the multiple-TRF1 organization of a single telomere. When controlling both TRF1 concentration and TL, the second-order partial correlation between *N* and Δ*L* shows *r* = –0.23 (*P* < 0.001, Supplementary Table S3). The negative correlation between the number of telomeric loops and the loop sizes may be explained by that TRF1 could compact a single telomere with primary loop domains to a high-order topology (Figure 4D).

### Strand separation of telomeric DNA drove the dissociation of TRF1

**Figure 5.**
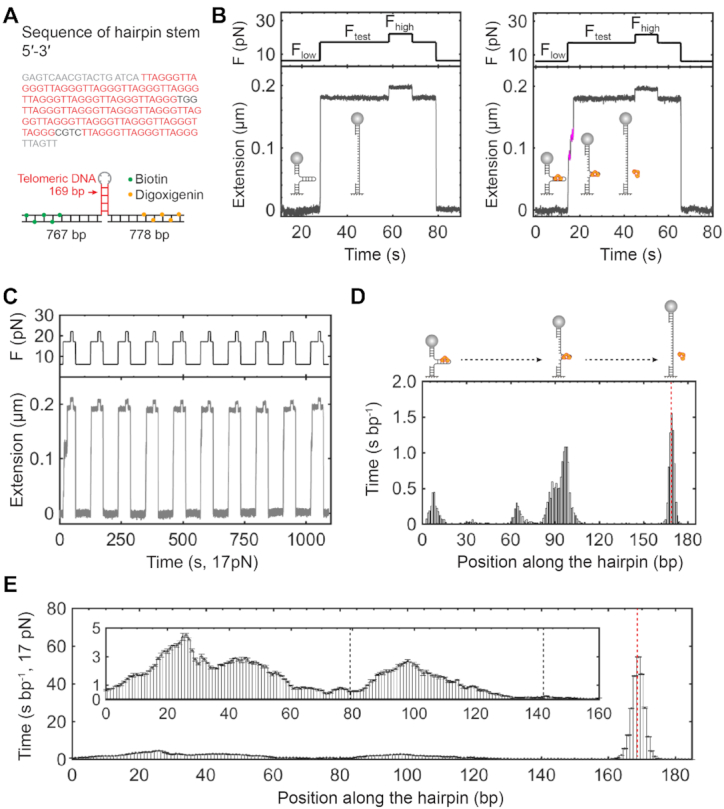
Sequential ruptures of TRF1–telomere complexes using strand-separation assays. (**A**) Telomeric hairpin construct with 23 repeats of TTAGGG. The telomeric hairpin construct contains a stem of 169 bp and two flanking handles (>700 bp) with either biotin or digoxigenin modifications (bottom). The G-rich strand sequence of the hairpin stem has 23 repeats of TTAGGG (red) with two spacers of non-telomeric DNA sequences (Black) and flanking sequences (gray) at both ends (top). (**B**) Individual strand-separation assays. Manipulation of forces at *F*_low_ = 6 pN, *F*_test_ = 17 pN and *F*_high_ = 22 pN determines the double strands of a telomeric hairpin stem to be either paired together or separated (left, cartoon). At the presence of TRF1 of 25 nM, bound proteins can interrupt the strand separation at *F*_test_ (right, cartoon). Pauses of extension reveal sequential rupture events with measurable dwell time (magenta). Buffer and temperature conditions are the same as those in Figure 3B. Sampling rate = 100 Hz. (**C**) Repetitive strand-separation assays. Incubation time at *F*_low_ is 60 s between two subsequent assays. (**D**) Site-specific time distribution of TRF1 dissociation based on a single trace of strand-separation assays. The red dashed line indicates the position where the hairpin is fully opened (cartoon). Bin size = 1 bp. (**E**) The averaged time distribution of TRF1 dissociation upon strand separation. The red dashed line indicates the position where the hairpins are fully open. Inset shows that two major valleys are distinguishable, as indicated by black dashed lines (mean ± SE, *n* = 173 from 10 molecules). The black dashed lines are corresponding to the positions of two non-telomeric sequences of the hairpin stem.

To further understand the role of TRF1 at telomeres upon fork movement, e.g. induced by replication or transcription, we designed a DNA hairpin construct for mechanical strand-separation assays. The hairpin stem has 23 repeats of TTAGGG motifs in 169 bp, close to dsDNA’s persistence length (47,58). The hairpin loop contains four nucleotides of thymine. At the fork end of the hairpin stem, there is a random sequence of 18 bp. We embedded two non-telomeric sequences, TGG and CGTC, in the stretch of the telomeric sequence after the 10th and 20th of TTAGGG motif, respectively (Supplementary Figure S6 and Figure 5A). The two non-telomeric sequences in the hairpin stem facilitate the correct base pairing in the strand-separation assays. Two strands of the telomeric dsDNA unzip when forces are above the critical melting force of 15.1 ± 0.6 pN (mean ± SD, *N* = 98) (Supplementary Figure S6). Because of the interactions between TRF1 and telomeric dsDNA, TRF1 can block the dsDNA strand separation from the upstream to the downstream. By probing interruptions of strand separation, we can site-specifically measure the dynamics of TRF1 dissociation from a telomeric DNA in real-time.

We used a protocol of three force levels in single-molecule mechanical strand-separation assays. At a low force (*F*_low_), such as 6 pN, the telomeric stem of the hairpin construct is in a dsDNA conformation (Figure 5B, left). At a testing force (*F*_test_) above the critical force of the telomeric stem, such as 17 pN, dsDNA unzips to be single-stranded DNA (ssDNA) cooperatively without pauses (Figure 5B, left). At a high force (*F*_high_), such as 22 pN, the melted telomeric stem further extends, assuring the complete separation of the hairpin stem. We then lowered down the force from *F*_high_ to *F*_test_, and *F*_low_, finishing one round of the protocol. At a TRF1 concentration of 25 nM in a buffer containing 20 mM of HEPES (pH 7.5), 1 mM of EDTA, 100 mM of NaCl and 0.0063% Tween-20, we performed the strand-separation assay using a protocol with *F*_low_ = 6 pN, *F*_test_ = 17 pN and *F*_high_ = 22 pN (Figure 5B, right). The pausing signals at *F*_test_ reveal the interruptions of strand separation by TRF1. The repetition of strand-separation assays allows us to statistically examine how TRF1 binds a telomeric dsDNA (Figure 5C).

We evaluated the site-specific time distribution of TRF1 dissociation upon strand separation. Taking the extension of the strand-separated telomeric stem at the *F*_test_, we can build a site-specific time histogram along with the ssDNA extension for each trace (Figure 5D). We obtained the overall time at each base after dividing the heights of histograms by the sampling rate of 100 Hz. Peak positions and heights reveal where TRF1 binds to its targeting motifs and how long it takes TRF1 to dissociate upon strand separation, respectively. From the averaged histogram of 173 traces (Figure 5E), we observed a complex landscape of TRF1 dissociation from a telomeric DNA. Along the telomeric sequence, two significant valleys are evident with bottoms at the two non-telomeric sites (TGG at the 79th bp and CGTC at the 142nd bp, respectively), showing that non-telomeric sequences can dramatically affect the interactions between TRF1 and a telomere.

### TRF1 dissociated in seconds from a (TTAGGG)_2_ site upon strand separation

**Figure 6.**
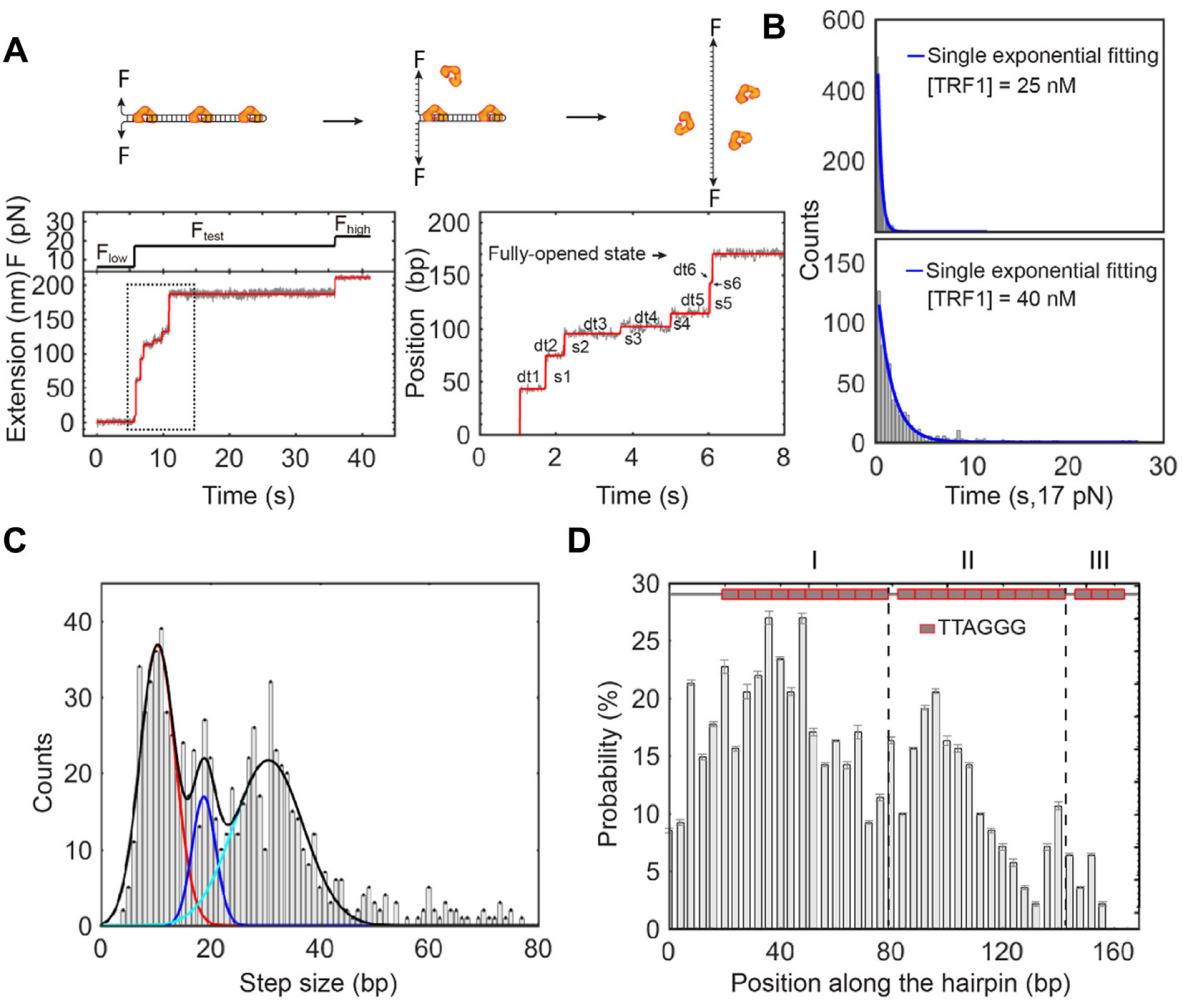
Analysis of dwell time, sub-step size and site-specific binding probability using a step-fitting algorithm. (**A**) A single trace of strand-separation assays modeled by a step-fitting algorithm. The red line is the result of the step-fitting algorithm (bottom left). The dotted frame is zoomed in to show the sub-step sizes (s1 ∼ s6) and dwell time (dt1–dt6) (Bottom right). The cartoon illustrates the sub-steps in a strand-separation assay (top). (**B**) Dwell time distribution of sub-steps at [TRF1] = 25 nM (top) and 40 nM (bottom). (**C**) Distribution of step sizes. The three-Gaussian curve (black) show a periodicity of ∼10 bp revealed by centers at 10 ± 3 bp (red), 19 ± 2 bp (blue) and 31 ± 6 bp (cyan) (estimate ± SD, *n* = 784 from 10 molecules). (**D**) Distribution of site-specific binding probability. The distribution shows three major domains as divided by two dashed lines (*N* = 784 from 10 molecules). The dashed lines match to the positions of non-telomeric sequences in the hairpin stem (cartoon).

We analyzed the dynamics of DNA–protein interactions to reveal that one sub-step indicates one dissociation event of a TRF1 dimer from the telomeric DNA in a strand-separation assay. We fitted every single trace of strand separation at *F*_test_ and [TRF1] = 25 nM using a step-fitting algorithm (48) (Figure 6A). We then collected the fitting results of the site, size, and duration for each sub-step (Figure 6A, bottom right). A single-exponential decay function is enough to describe the distribution of dwell time (0.946 for the *R*-squared value) and reveals that τ = 0.36 ± 0.02 s at [TRF1] = 25 nM (estimate ± SE, and *N* = 1290) (Figure 6B, top). We also did strand-separation assays at a high concentration of [TRF1] = 40 nM and found a single-exponential coefficient of τ = 1.71 ± 0.09 s (estimate ± SE, *N* = 588, and 0.903 for the *R*-squared value) (Figure 6B, bottom). The longer time coefficient at [TRF1] = 40 nM than that at 25 nM can be explained by the overall higher binding energies from more binding TRF1 proteins (Figure 2D). Since the hairpin stem used here is close to the persistence length of dsDNA and shorter than the TRF1-compacted loops of ∼250 bp as discovered above, the telomeric dsDNA most likely remains a linear conformation without compaction.

Analysis of the step size distribution reveals a periodicity of TRF1 binding telomeric dsDNA. We found that a three-Gaussian function describes the distribution the best with the peaks at 10 ± 3, 19 ± 2 and 31 ± 6 bp (estimate ± SD, *n* = 784), resulting in a periodic unit of ∼10 bp (Figure 6C). Because a TRF1 dimer binds two neighboring motifs of TTAGGGTTAGGG, 12 bp (9), the agreement within one standard deviation between our measurements and the structure-based prediction suggests that TRF1 binds as a dimer configuration on a telomere. Strand separation thus drives TRF1 away one dimer at a time and releases a unit length of ∼10 bp.

Furthermore, we analyzed the binding probabilities of TRF1 along a stretch of telomeric dsDNA upon strand separation. The distribution of TRF1 binding probabilities shows three domains along the telomeric dsDNA (Figure 6D). The domain boundaries are around the positions of non-telomeric sequences, 79th bp and 142nd bp, as mentioned above (Figure 5E). From the upstream to the downstream, domain I-III reveals a unidirectional decrease of TRF1 binding probabilities, which may be explained by the asymmetrical scenario of strand separation. The fork-like DNA hairpin construct has two flanking dsDNA handles for mechanical manipulation. The nonspecific binding of protein on dsDNA handles has a rate exceeding the diffusion-limited *k*_on_ (66). During the target search, TRF1 can bind dsDNA handles nonspecifically and laterally diffuse along dsDNA until it finds on a target site (67,68). Fork handles of dsDNA may thus attract TRF1 and provide a local pool of proteins, which causes the unidirectionally decreased binding probability along the telomeric hairpin stem.

### TRF1 can temporarily reverse the strand separation of telomeric dsDNA

**Figure 7.**
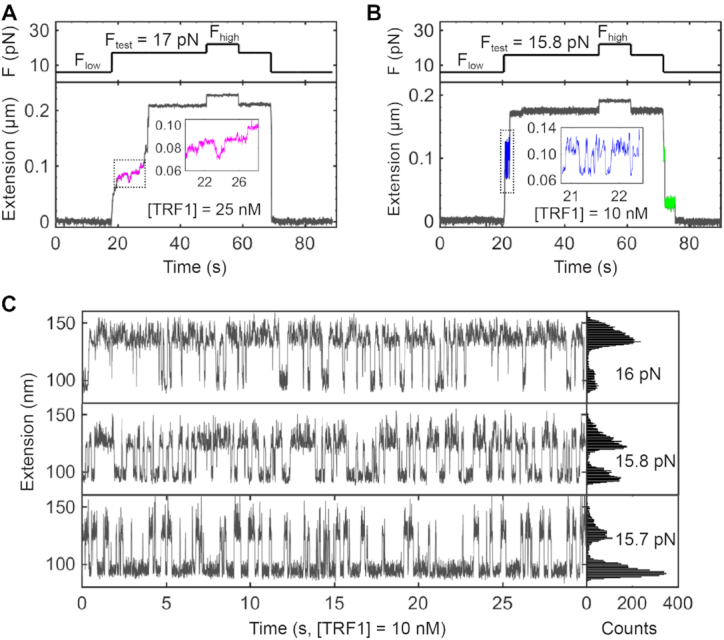
Reversed steps in strand-separation assays at the presence of TRF1. (**A**) Reversed steps upon strand separation. Reverse steps occasionally happen at *F*_test_ = 17 pN with [TRF1] = 25 nM (magenta). The dotted frame is zoomed in to show reverse steps in detail (inset). (**B**) Hopping steps upon strand separation. Backward and forward steps hop at *F*_test_ = 15.8 pN with [TRF1] = 10 nM (blue). The dotted frame is zoomed in to show hopping steps in detail (inset). When finishing a strand-separation assay, changes in extension pauses at the transition from *F*_test_ to *F*_low_ (green), which is not observed in (A). (**C**) Hopping events modulated by *F*_test_. At the concentration of [TRF1] = 10 nM, small changes in *F*_test_ (15.7, 15.8 and 16 pN) modulate the equilibrium of hopping events to distinct distributions of backward and forward states. Buffer and temperature conditions are the same as those in Figure 3B. Sampling rate = 100 Hz in (A), 200 Hz in (B) and (C).

While the forward movement of a fork drives the dissociation of TRF1 upon strand separation, we occasionally observed backward steps of fork movements at *F*_test_ = 17 pN. Reverse steps suggest that the fork of the telomeric hairpin stem moves backward due to the blockage by a TRF1 binding event (Figure 7A). To further examine the backward steps, we next modulated the *F*_test_ from 17 pN down to 15.8 pN, which is still >1 standard deviation higher than the critical force of 15.1 ± 0.6 pN (mean ± SD, *N* = 98). Also, we decreased the TRF1 concentration from 25 to 10 nM, crossing the *K*_d_ of 17.5 nM and reducing the binding events in a single strand separation assay. At *F*_test_ = 15.8 pN and [TRF1] = 10 nM, the occasionally happened backward steps become hopping events with high frequency, indicating that TRF1 blockage on the strand separation of a telomeric dsDNA is sensitive to forces and protein concentrations (Figure 7B, inset). Without TRF1, strand-separation assays show no pauses when forces jumped from *F*_low_ = 6 pN to *F*_test_ = 15.8 pN, or vice versa. On the other side, many pauses happen upon a force jump from *F*_test_ = 15.8 pN to *F*_low_ = 6 pN at the presence of TRF1, which contrasts to the single drop of extensions with a force jump from 17 to 6 pN (Figure 7A versus B, green). Both hopping events and pauses mentioned above manifest that TRF1 binding events can interrupt the unzipping and zipping processes of telomeric dsDNA.

Upon the blockage of TRF1, forces experienced by the DNA fork can fine-tune the dynamics between double-stranded and single-stranded conformations. During the process of strand separation at a testing force of 15.7 pN and [TRF1] = 10 nM, the DNA equilibrates between locally forward and backward states (Figure 7C, bottom). The overall time is longer at the backward state than that at the forward state. When the testing force increases to 15.8 pN by 0.1 pN, the telomeric DNA shows equally distributed time for the two states of forward-backward equilibrium (Figure 7C, middle). A testing force of 16 pN, 0.2 pN stronger than 15.8 pN, tunes the time to be longer at the forward state than that at the backward state (Figure 7C, top). The interplay among TRF1, telomeric DNA, and forces reveals that TRF1 plays a role in telomere strand separation, a scenario frequently happening in, e.g. replication, transcription, and DNA-damage responses.

### TRF1 modulated the fast dynamics of a telomeric fork with strong binding energies

To characterize the role of TRF1 in modulating the dynamics of a telomeric fork, we designed a short hairpin construct that contains 13 repeats of TTAGGG in the stem. The short telomeric hairpin had a similar configuration as that of the long hairpin construct with 23 repeats of the TTAGGG motif (Supplementary Figure S7). However, the short DNA allows us to observe hopping events upon the fork movement, which are rare in the long one. Under the same buffer conditions as that for the long hairpin construct, we performed strand-separation assays at constant forces using the short hairpin construct.

**Figure 8.**
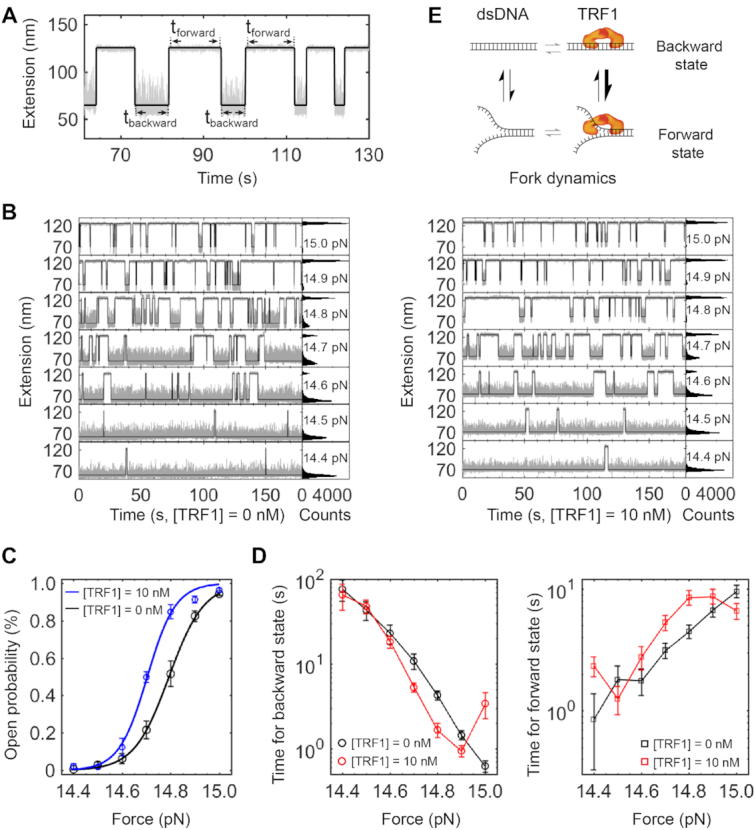
TRF1 modulation of a telomeric DNA fork. (**A**) Dwell time measurements on a hopping trace. A two-state Hidden Markov Model (HMM) (Black) reveals the dwell time of t_forward_ and t_backward_. Buffer and temperature conditions are the same as those in Figure 3B. Sampling rate = 200 Hz. (**B**) HMM analysis of hopping traces at various *F*_test_. The concentration of TRF1 is 0 nM for the left panels and 10 nM for the right panels, respectively. (**C**) The open probability of a telomeric fork as a function of forces. Curves represent fittings to a Boltzmann relation model. (**D**) The dwell time on forward and backward states as a function of forces. (**E**) Scheme illustrating the TRF1 effect on the dynamics of a telomeric DNA fork.

We ran hidden Markov modeling (HMM) to characterize the fork dynamics at constant forces (49,50). At a constant force of 14.8 pN, which can locally move the DNA fork by partially separate two strands, we observed that the telomeric DNA hops between forward and backward states at the absence of TRF1 (Figure 8A). Fitted to an HMM model of two states, we revealed the forward and backward states in the telomeric DNA fork dynamics. We further fine-tuned the force from 14.4 to 15 pN by a step size of 0.1 pN in strand-separation assays. Fork dynamics of the telomeric DNA can reach equilibrium at each force (Figure 8B, left). However, time distributions at the forward and backward states evolve in opposite directions when forces increase. We next performed the strand-separation assays at [TRF1] = 10 nM under the same experimental conditions as above. After HMM analysis, we collected the durations for each interconversion between the forward and backward states (Figure 8B, right).

To better understand the dynamics of the interactions between TRF1 and a telomeric fork, we here estimated the free energies using a simple two-state model with a single energy barrier. We quantified the populations of forward open state (*P*_o_) as a function of forces (*F*), which follows the Boltzmann relation (Figure 8C) (69). Using the Boltzmann relation, ${P_o}( F ) = {( {1 + {\rm{exp}}( {{F_{1/2}} - F} ) \cdot \Delta x} )^{ - 1}}$, we obtained the unfolding distance (Δx) and unfolding force (F_1/2_) at which the telomeric fork is fully open at a probability of 50%. Because Δ*G* = *F*_1/2_·Δ*x*, we estimated the changes in free energies to be 61 *k*_B_*T* and 50 *k*_B_*T* with and without TRF1, respectively. Hence, the ΔΔG due to the TRF1 binding effect is 11 *k*_B_*T*, which is similar to the energy required by chromatin-remodellers to break a DNA–histone contact, 12.3 *k*_B_*T* (70).

Interestingly, we found that TRF1 makes the dwell time at the backward state 8.7 s shorter at *F*_1/2_ = 14.7 pN than that without TRF1 (Figure 8D, left). On the other side, dwell time at the forward state becomes 1.6 s longer at *F*_1/2_ = 14.7 pN and [TRF1] = 10 nM than that without the protein (Figure 8D, right). Around the forces generating evenly distributed extension of two states, the modulation effect of dwell time at the DNA fork by TRF1 is more evident than that at forces giving severely biased extension distributions. The results above reveal that TRF1 can locally modulate the dynamics of DNA fork upon partial strand separation, which generates time windows for enzyme activities (Figure 8E).

## DISCUSSION

Using single-molecule techniques, we have investigated the dynamics of TRF1 in organizing a single human telomere. We first established a method for mechanically manipulating a single human telomere. From K562 cells, we prepared single human telomeres that carried digoxigenin and biotin modifications at both ends for single-molecule force–extension assays. Our single-molecule method provides the direct measurements of telomere length (TL), which can be analytically evaluated by a WLC model (55,57–59). The TL measurements by our single-molecule method are highly precise at the bp resolution. Our TL method based on single-molecule force spectroscopy measures one telomere at a time without signal amplification and gives a continuous distribution, which is in contrast to the rough estimation by TRF, PCR or fluorescence (38,61).

Methods of TL measurement based on gel imaging analysis of PCR products suffer from a strong background and low contrast for the short telomeres. By directly measuring TL one molecule at a time, the single-molecule method can circumvent the background issue of gel imaging analysis and reveal the length distribution of telomeres, especially the short ones at any defined length ranges. Extension measurements using single-molecule force spectroscopy reveals the shortest TLs at a low occurring frequency. The short telomeres <1 kb occurs 10× less than the long ones peaked at 2.5 kb. Because the short telomeres can activate DNA damage responses and cellular senescence, the capability of measuring the shortest telomeres without background noise like that in gel imaging of PCR products demonstrates that single-molecule force spectroscopy can serve as a unique tool in the research of telomere biology, epidemiology, and cancer therapy, among others.

Compaction and decompaction of a telomere are essential for chromatin during cell division (16). Instead of nucleosomes, shelterin proteins play a critical role in organizing a single telomere (5). In this work, both force–extension and force–jump assays reveal that a TRF1 dimer can compact a single human telomere by interacting with two targeting sites separated far apart. The distance between two sites can be as far as 2784 bp, suggesting that TRF1 can quickly condense a human telomere by forming large loops. At a stretching force of 2–8 pN, we found that TRF1–DNA complex ruptured in tens of seconds, revealing that TRF1 has a large time window for interfering telomere activities, e.g. replication, transcription, and DNA damage responses (36,71,72). Telomere volume measured by super-resolution microscopy shows controversial results of shelterin compacting telomeres in a cell (6,31,32). Our bottom-up method of single telomere compaction assay allows us to dissect the condensation event by adding shelterin subunits one by one, providing a new strategy to investigate how shelterin proteins protect chromosomes from a perspective of mechanics and kinetics.

Meanwhile, we found that non-telomeric sequences can dramatically affect the interactions between TRF1 and a telomere. Both dissociation time and binding probabilities of TRF1 on an artificial telomere drop to form valleys around the positions of non-telomeric sequences. This finding helps to explain the reason why a human telomere contains continuous repeats of TTAGGG motifs without interruptions and why mutations in TTAGGG repeats form a well-isolated region of subtelomere. Our finding also suggests that non-telomeric linker sequences in artificial telomere constructs should be under careful consideration when interpreting the data. Instead of making artificial telomeric dsDNA constructs by conventional methods with plasmids, we have directly obtained the single human telomeres from cells, which thus provides non-biased results in studying the TRF1’s compacting function.

The molecular architecture of TRF1–DNA complexes requires both protein–protein interactions and protein–DNA interactions (9,16,63,73). We have stretched a single compacted telomere to reveal looped structures mediated by protein–protein interactions when TRF1 forms homodimers to condense DNA. On the other hand, the direct interactions between TRF1 and DNA play a pivotal role in TTAGGG repeat-associated replication, averting TRF2-dependent telomeric R loops, and telomere breakages (25,28,29,74). The TTAGGG repeats of telomeres challenge the DNA replication machinery, resulting in replication-dependent defects (29,75,76). Gene deletion assays and single-molecule fluorescence experiments of replicating telomeres reveal that TRF1 is essential for efficient replication of mammalian telomeres by preventing fork stalling (29). We examined the dynamics of TRF1 at a telomeric DNA fork using single-molecule strand-separation assays. We found that TRF1 can modulate the forward and backward steps of DNA fork movements by 2–9 s at a critical force of *F*_1/2_, generating an overall effect of maintaining the telomeric fork at an open state. Considering that DNA replication usually happens at a rate of >100 bp/s (72,77), our results from the telomeric fork assay provide insights into the kinetics of how TRF1 facilitated the efficient replication of a telomere. The experimental methods we developed here could be potentially used for further investigation on telomere replication. Our methods and results will also help future research on TRF1 as a drug target (78–80).

## Supplementary Material

gkaa1222_Supplemental_File
