# Supplementary material for: Dynamics of TRF1 organizing a single human telomere
Source: Nucleic Acids Res. 2020 Dec 21;49(2):760–75. doi: 10.1093/nar/gkaa1222 (PMC7826288; doi:10.1093/nar/gkaa1222)
Supplement: gkaa1222_Supplemental_File [file gkaa1222_supplemental_file.pdf]

Dynamics of TRF1 Organizing a Single Human Telomere

Xu Li[1], Meijie Wang[1], Wei Zheng[1], Wei Huang[1], Zeyu Wang[1], Kairang Jin[2], Lin Liu[2], Zhongbo Yu[1]*

[1] State Key Laboratory of Medicinal Chemical Biology, College of Pharmacy, Nankai University, 38 Tongyan Road, Tianjin 300350, China

[2] State Key Laboratory of Medicinal Chemical Biology, Department of Cell Biology and Genetics, College of Life Sciences, Nankai University, 94 Weijin Road, Tianjin 300071, China

* To whom correspondence should be addressed. Tel: +86 22 8535 8335; Fax: +86 22 8535 8291; Email: zyu@nankai.edu.cn

This Supplementary Information includes:

Table S1. DNA oligos

Table S2. The amino acid sequence of TRF1

Table S3. Correlation coefficients between variable pairs measured in telomere-compacting assays

Figure S1. Integrity examination of genomic DNA

Figure S2. Assessment of non-telomeric DNA using dot-blot analysis

Figure S3. Comparison of force-extension curves with and without TRF1

Figure S4. Force-jump assays to measure the dissociation time of TRF1 from telomeric dsDNA

Figure S5. Correlation analysis of the variables measured in telomere-compacting assays

Figure S6. Construction and characterization of the long telomeric hairpin

Figure S7. Construction and characterization of the short telomeric hairpin

Table S1. DNA oligos

Name	Sequence (5′-3′)
Junction 1	tcagcAAGGAAGGAGATTTTGAAAAATTTATTTATTAGATATTG<br>GAAATATTATTAGAGGAGATGATTTAAAAAAATATGAAGAAT<br>GGTATAATAAAAGGgTTTgagtcaacgtactg
Junction 2	cCCTTTTATTATACCATTCTTCATATTTTTTTAAATCATCTCCT<br>CTAATAATATTTCCAATATCTAATAAATAAATTTTTCAAAATCT<br>CCTTCCTTgc
Junction 3	tgatcagtacgttgactcTTTgGGAGTAGATGTGGTTTTTGTTTTTT<br>TGAATAATAAATGTTAAAAAAAGTGGGGAAGTGAGTAATGA<br>AATTATTTTGTATGTTTTTTATATGAATTTATTTTTTGgg
Junction 4	GACccCAAAAAATAAATTCATATAAAAAACATACAAAATAATT<br>TCATTACTCACTTCCCCACTTTTTTTAACATTTATTATTCAAA<br>AAAACAAAAACCACATCTACTCCc
Stem 1	ATCATTAGGGTTAGGGTTAGGGTTAGGGTTAGGGTTAGGG<br>TTAGGGTTAGGGTTAGGGTTAGGGTGG
Stem 1c	CCCTAACCCTAACCCTAACCCTAACCCTAACCCTAACCCTA<br>ACCCTAACCCTAACCCTAA
Stem 2	TTAGGGTTAGGGTTAGGGTTAGGGTTAGGGTTAGGGTTAG<br>GGTTAGGGTTAGGGTTAGGG CGTC
Stem 2c	CCCTAACCCTAACCCTAACCCTAACCCTAACCCTAACCCTA<br>ACCCTAACCCTAACCCTAACCA
Loop 1	TTAGGGTTAGGGTTAGGGTTAGTTTTCTAACCCTAACCCTA<br>ACCCTAAGACG
Loop 2	TTAGGGTTAGGGTTAGGGTTAGTTTTCTAACCCTAACCCTA<br>ACCCTAACCA
Forward primer for biotin handle	GACCGAGATAGGGTTGAGTG
Reverse primer for	gcatcggctgagg AAAGGGAACAAAAGCTGG

biotin handle	
Forward primer for Digoxigenin handle	ATCGTAGGGTCCTGACCGAGATAGGGTTGAGTG
Reverse primer for Digoxigenin handle	AAAGGGAACAAAAGCTGG
Forward primer for TRF1$^{3\text{-}439}$	CGGGATCCGAGGATGTTTCCTCAGCGGC
Reverse primer for TRF1$^{3\text{-}439}$	CCGCTCGAGTCAGTCTTCGCTGTCTGAGG

Table S2. The Amino acid sequence of TRF1

Name	Sequence (3-439)
TRF1	EDVSSAAPSPRGCADGRDADPTEEQMAETERNDEEQFECQ<br>ELLECQVQVGAPEEEEEEEEDAGLVAEAEAVAAGWMLDFLC<br>LSLCRAFRDGRSEDFRRTRNSAEAIIHGLSSLTACQLRTIYICQ<br>FLTRIAAGKTLDAQFENDERITPLESALMIWGSIEKEHDKLHEE<br>IQNLIKIQAIAVCMENGNFKEAEEVFERIFGDPNSHMPFKSKLL<br>MIISQKDTFHSFFQHFSYNHMMEKIKSYVNYVLSEKSSTFLMK<br>AAAKVVESKRTRTITSQDKPSGNDVEMETEANLDTRKSVSDK<br>QSAVTESSEGTVSLLRSHKNLFLSKLQHGTQQQDLNKKERR<br>VGTPQSTKKKKESRRATESRIPVSKSQPVTPEKHRARKRQA<br>WLWEEDKNLRSGVRKYGEGNWSKILLHYKFNNRTSVMLKDR<br>WRTMKKLKLISSDSED

Table S3. Correlation coefficients between variable pairs measured in telomere compacting assays

Controlled variables		N	ΔL	TL	Conc
Conc	N	1.00			
	ΔL	-0.19***	1.00		
	TL	0.13***	0.27***	1.00	
TL	N	1.00			
	ΔL	-0.24***	1.00		
	Conc	0.13***	-0.06*		1.00
Conc and TL	N	1.00			
	ΔL	-0.23***	1.00		

Note: N = 1496 from 131 molecules. *: $p < 0.05$. ***: $p < 0.001$.

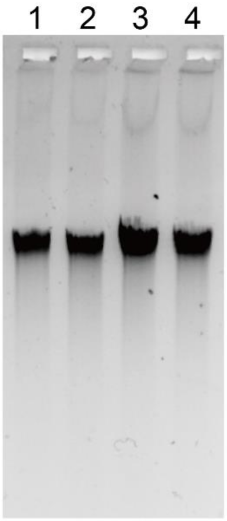

Figure S1. Integrity examination of genomic DNA

Electrophoresis of four parallel DNA samples (400 ng each) was done in 1% agarose gel at 150 V for 30 min. The four bands migrate in parallel and appear as compact crowns, indicating that all the genome samples contain DNA in good integrity.

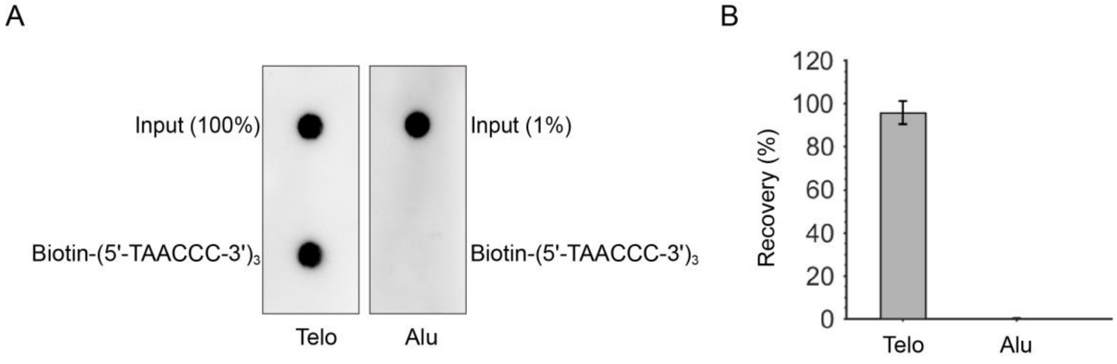

Figure S2. Assessment of non-telomeric DNA using dot-blot analysis

(A) Dot blot of DNA before and after enrichment with either a telomere-specific probe (Telo: dig-(5′-CCCTAA-3′)$_3$) or a probe against Alu-repeat DNA (Alu: dig-(5′-GTGATCCGCCCGCCTCGGCCTCCCAAAGTG-3′)). 100% DNA (20 μg) was loaded for the detection except that the input for Alu probing used 1% of total DNA (0.2 μg).

(B) Recovery quantification of the dot-blot represented in (A). Error bars represent standard deviations of three independent experiments.

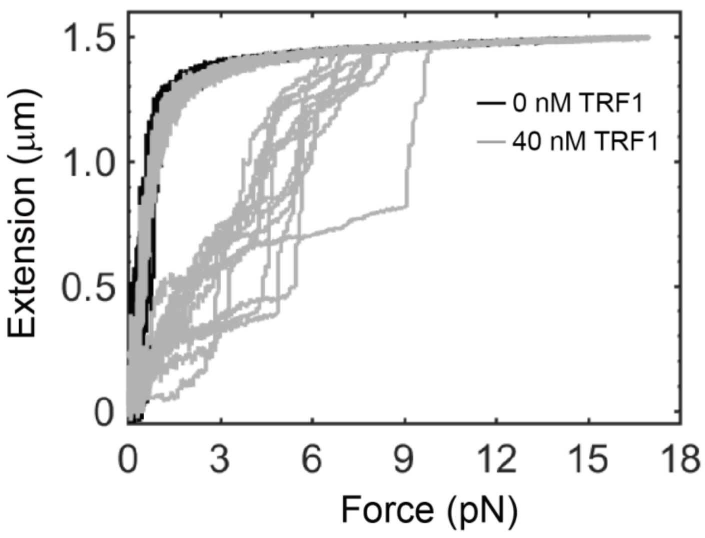

Figure S3. Comparison of force-extension curves with and without TRF1

The grey data are force-extension curves for telomeric DNA at [TRF1] = 40 nM (N = 13) and the black data are traces at [TRF1] = 0 nM (N = 8). Forces oscillate between 0 and 17 pN at a loading rate of ± 4 pN/s. The buffer contains 20 mM of HEPES (pH 7.5), 1 mM of EDTA, 100 mM of NaCl and 0.0063% Tween-20. Sampling rate = 200 Hz.

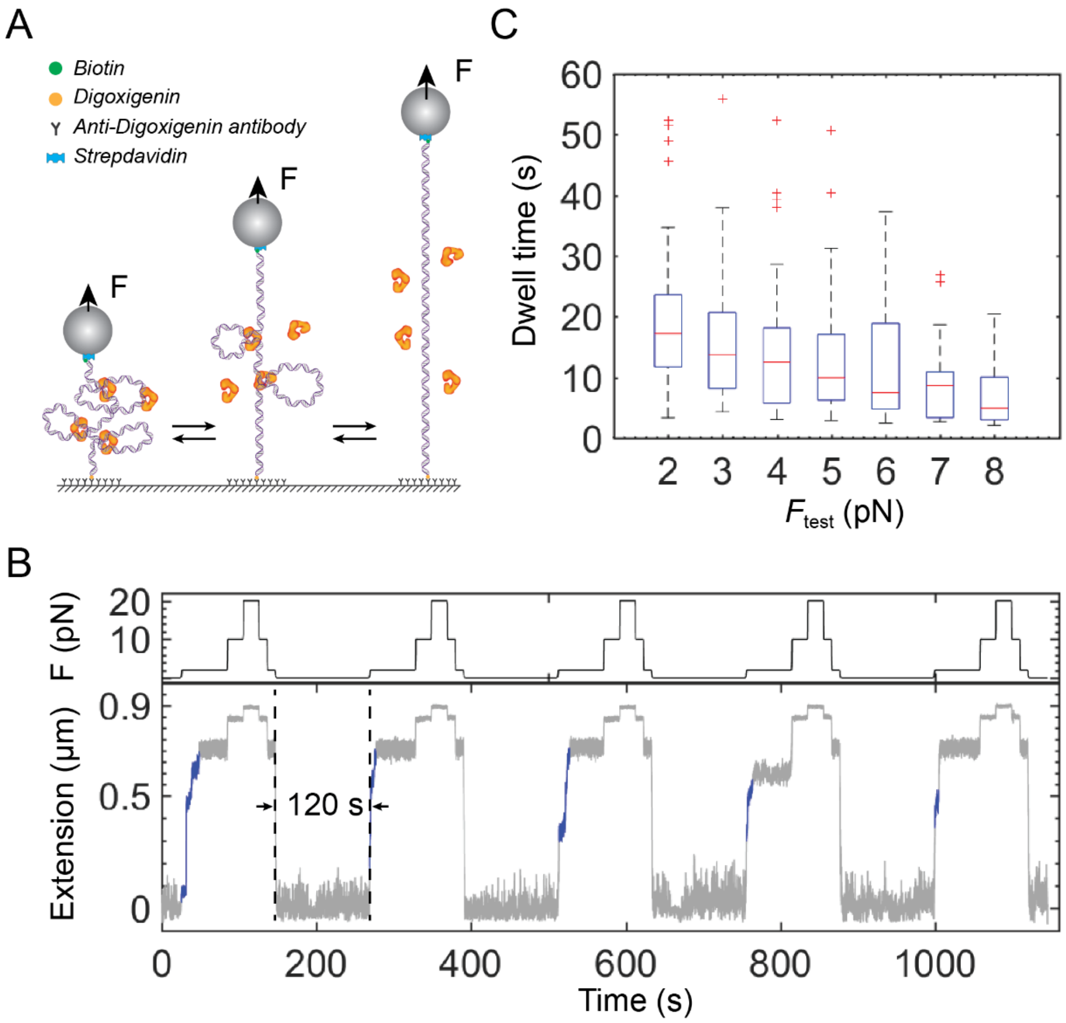

Figure S4. Force-jump assays to measure the dissociation time of TRF1 from telomeric dsDNA

(A) Scheme of the experimental setup.

(B) Repetitive force-jump assays. Incubation time is 120 s between two subsequent assays. Blue color highlights the pausing signals upon changes in extension.

(C) Dwell time of TRF1 on a single human telomere upon forces (N = 206). Red bar: Median. Box edges: the 25th and 75th percentiles. Whiskers: the 9th and 91st percentiles. Plus sign: Outliers of data.

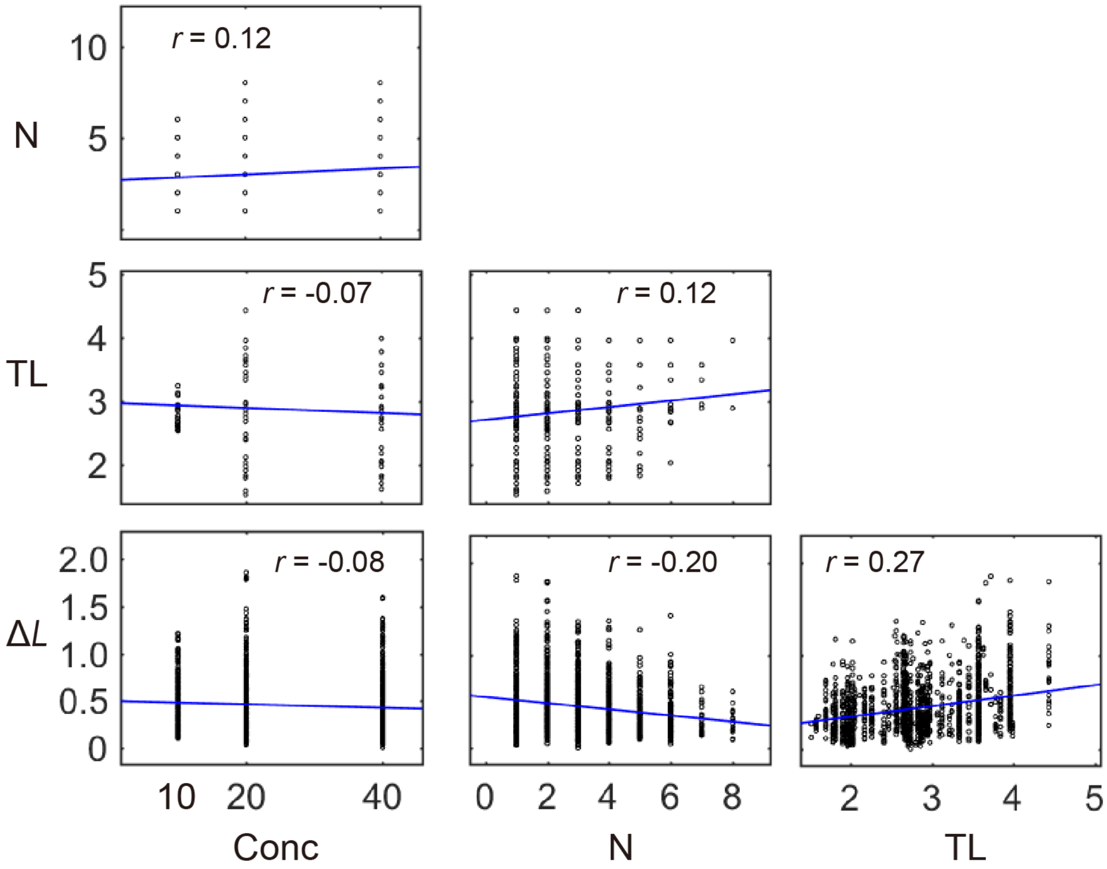

Figure S5. Correlation analysis of the variables measured in telomere-compacting assays

Zero-order coefficients for Pearson's correlation are noted in each panel with all the p values < 0.001 except that p values for TL vs. Conc and Δ*L* vs. Conc are < 0.01.

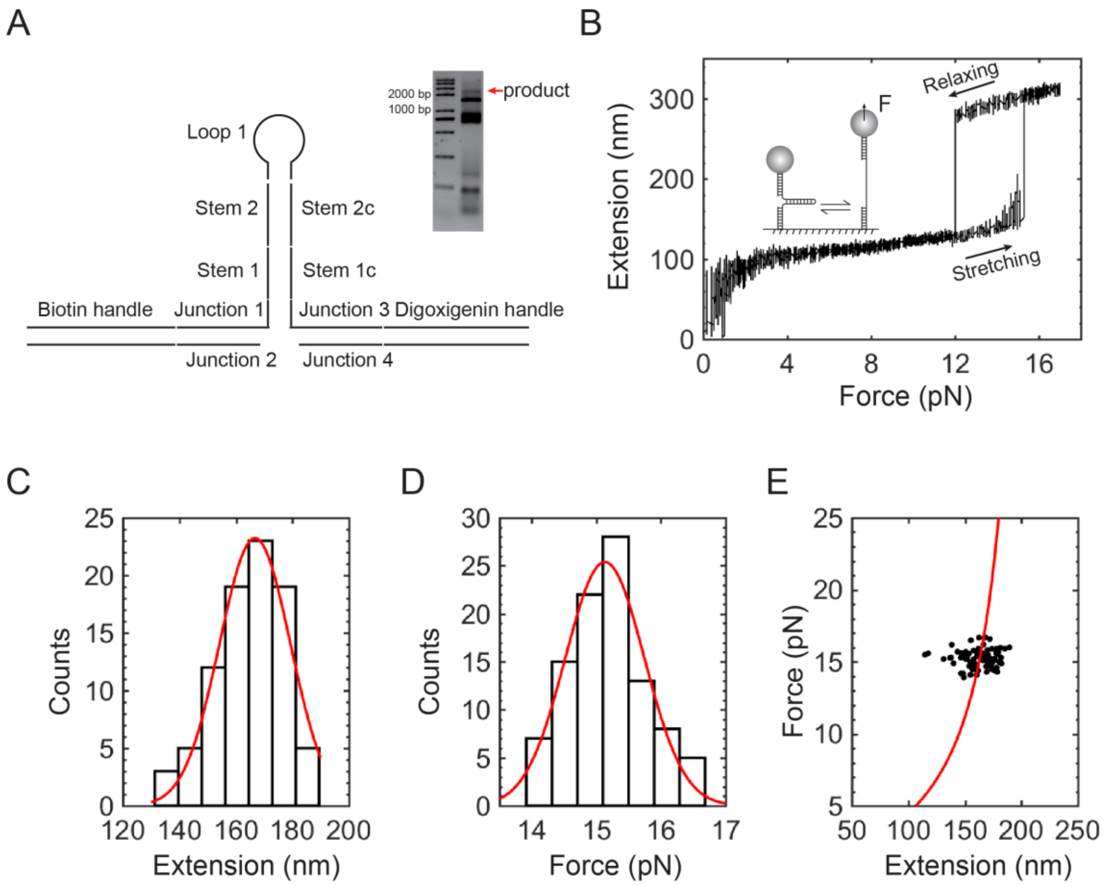

Figure S6. Construction and characterization of the long telomeric hairpin

(A) Scheme for construction of a long telomeric hairpin. Inset shows the final product after ligation (1% agarose gel electrophoresis).

(B) Force-extension assay to examine the processes of telomeric DNA unzipping and rezipping.

(C) Distribution of changes in extension for DNA unzipping events. The red curve represents a Gaussian fit centered at 167 ± 13 nm (Estimate ± sd, n = 86).

(D) Distribution of unzipping forces. The red curve represents a Gaussian fit centered at 15.1 ± 0.6 pN (Estimate ± sd, n = 98).

(E) A plot of force vs. changes in extension. Black dots are the same data as that in (C) and (D). The red line is an estimation from a WLC model.

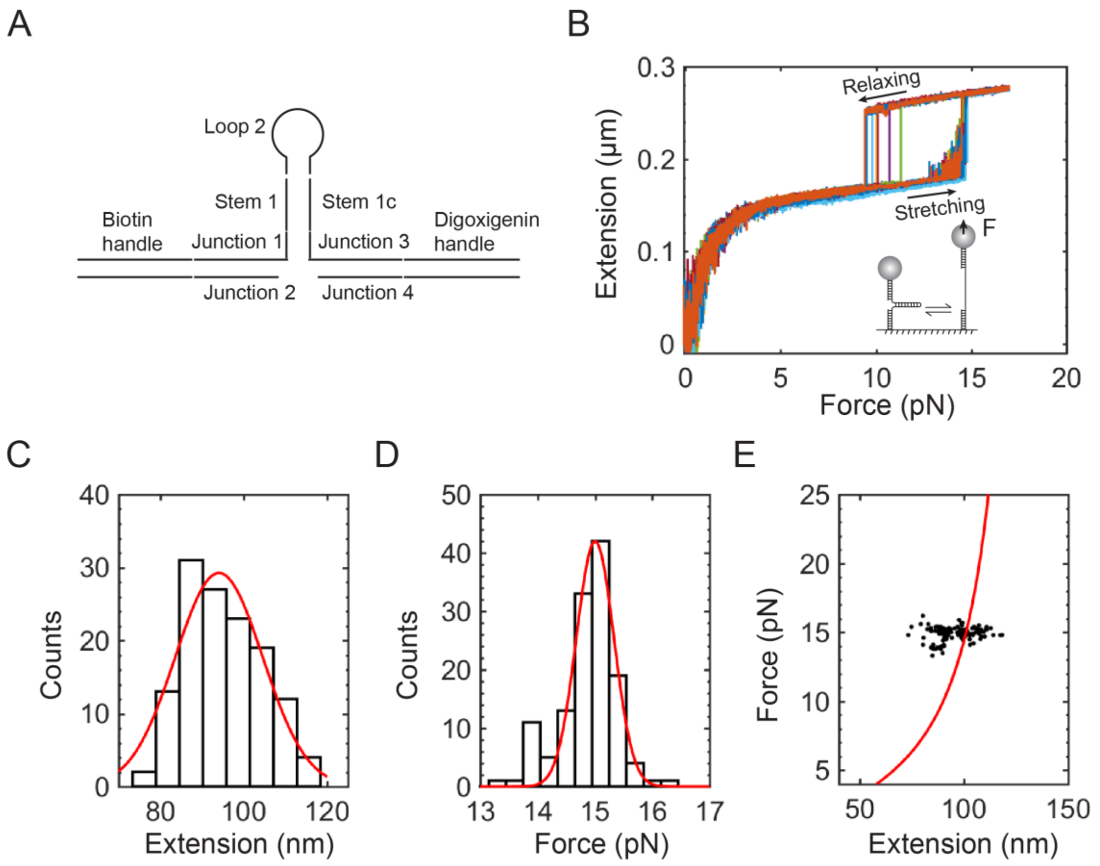

Figure S7. Construction and characterization of the short telomeric hairpin

(A) Scheme for construction of a short telomeric hairpin.

(B) Force-extension assays to examine the processes of DNA unzipping and rezipping.

(C) Distribution of changes in extension for DNA unzipping events. The red curve represents a Gaussian fit centered at 94 ± 10 nm (Estimate ± sd, n = 131).

(D) Distribution of unzipping forces. The red curve represents a Gaussian fit centered at 15.0 ± 0.3 pN (Estimate ± sd, n = 131).

(E) A plot of force vs. changes in extension. Black dots are the same data as that in (C) and (D). The red line is an estimation of a WLC model.
